# ZBP1 activated by mitochondrial damage binds to STAT3 to suppress Legumain production and exacerbate macrophage inflammation in *Klebsiella pneumoniae*-induced sepsis

**DOI:** 10.1016/j.mmr.2026.100059

**Published:** 2026-07-30

**Authors:** Li-Ting Deng, Huan Yang, Xu-Fei Zhang, Ze-Xing Lin, Ni Kuang, Hai-Yang Jiang, Pei-Zhao Liu, Yang-Guang Li, Xuan-Heng Li, Wei-Zhen Li, Chu-Jun Ni, Ming-Jie Qiu, Yue Chao, Yu-Fei Pan, Yi-Yu Yang, Ye-Ting Zhu, Yun Zhao, Jian-An Ren, Xiu-Wen Wu

**Affiliations:** a School of Medicine, Southeast University, Nanjing 210009, China; b Research Institute of General Surgery, Jinling Hospital, Affiliated Hospital of Medical School, Nanjing University, Nanjing 210002, China; c Department of General Surgery, Clinical Translational Research Center for Surgical Infection and Immunity, the Affiliated BenQ Hospital of Nanjing Medical University, Nanjing 210026, China; d Department of General Surgery, Nanjing Drum Tower Hospital, Affiliated Hospital of Medical School, Nanjing University, Nanjing 210008, China; e Department of Infectious Diseases, Chongqing Key Laboratory for Research of Infectious Diseases, Southwest Hospital, Army Medical University, Chongqing 400038, China; f Department of Emergency Surgery, the Affiliated Hospital of Qingdao University, Qingdao 266000, Shandong, China; g Nanjing Medical University, Nanjing 210000, China; h Nanjing Medical Key Laboratory of Laryngopharynx-Head and Neck Oncology, the Affiliated BenQ Hospital of Nanjing Medical University, Nanjing 210026, China

**Keywords:** *Klebsiella pneumoniae* (*KP*), Z-DNA binding protein 1 (ZBP1), Signal transducer and activator of transcription 3 (STAT3), Sepsis, Macrophage, Legumain (LGMN)

## Abstract

**Background:**

Severe bacterial sepsis caused by *Klebsiella pneumoniae* (*KP*) is characterized by dysregulated inflammation, multiorgan injury, and high mortality, yet the key molecular drivers of this process remain incompletely understood. This study aimed to elucidate how the Z-DNA binding protein 1 (ZBP1) regulates inflammatory injury during *KP*-induced sepsis and to identify potential therapeutic targets with translational relevance.

**Methods:**

To investigate the role of macrophage ZBP1 in sepsis, we combined transcriptomic datasets from human sepsis cohorts and murine sepsis and *KP*-infection models with single-cell RNA sequencing of infected tissues. We further established both global and myeloid-specific *Zbp1* knockout (*Zbp1*^*fl/fl*^*Lyz2-Cre*^*+/-*^) mice, as well as myeloid-specific *Lgmn* (encoded Legumain) knockout (*Lgmn*^*fl/fl*^*Lyz2-Cre*^*+/-*^, *Lgmn*^*CKO*^) mice, to delineate the macrophage-dependent mechanisms of immune regulation. In addition, macrophage-targeted adeno-associated virus 9 (AAV9) vectors driven by the *F4/80* promoter were used to overexpress *Lgmn in vivo*. Flow cytometry, immunofluorescence, and survival analyses were performed to evaluate systemic inflammation, organ injury, and sepsis severity. The effect of recombinant LGMN supplementation on macrophage activation and barrier restoration was also assessed.

**Results:**

ZBP1 activation was driven by both type I interferon (IFN-I) signaling and mitochondrial damage. Mechanistically, ZBP1 directly interacted with signal transducer and activator of transcription 3 (STAT3), suppressed its phosphorylation and nuclear translocation, and consequently inhibited transcription of *Lgmn*, a gene associated with anti-inflammatory macrophage polarization and tissue repair. Genetic and myeloid-specific deletion of *Zbp1* resulted in reduced proinflammatory cytokine production and improved survival in both *KP-* and cecal ligation and puncture (CLP)-induced sepsis models. In contrast, *Lgmn*^*CKO*^ aggravated systemic inflammation, organ injury, and barrier dysfunction, whereas macrophage-targeted AAV9-mediated *Lgmn* overexpression or recombinant LGMN supplementation alleviated macrophage inflammation, restored epithelial function, and improved survival.

**Conclusion:**

These findings reveal a previously unrecognized ZBP1-STAT3-LGMN signaling axis that contributes to immune and inflammatory dysregulation in *KP*-induced sepsis and suggest that targeting ZBP1 or restoring LGMN activity may represent a promising therapeutic strategy for severe bacterial infections.

## 1 Background

*Klebsiella pneumoniae* (*KP*) is an encapsulated bacterium that accounts for approximately 27% of Gram-negative infections in intensive care units and causes both community-acquired and nonsocomial infections, ranging from pneumonia and urinary tract infections to bacteremia and sepsis 1, 2, 3, 4. Over the past decades, the emergence of hypervirulent *KP* (hv*KP*) and carbapenem-resistant strains has greatly expanded the clinical burden associated with *KP*, as these variants can infect immunocompromised individuals and cause severe, often fatal infections even in otherwise healthy hosts 5, 6, 7. A recent study indicates that the high mortality associated with hv*KP* is not merely due to bacterial proliferation, but rather stems from the overwhelming host immune response [8]. This cytokine storm-like response is especially evident in sepsis models, where systemic inflammation rapidly progresses to multi-organ dysfunction 9, 10. Despite extensive research on the antimicrobial resistance and immune evasion strategies of *KP*, the molecular mechanisms by which the host-pathogen interaction escalates into uncontrolled inflammation remain incompletely understood.

Macrophages are central effectors of the innate immune response during bacterial infections, particularly those caused by Gram-negative pathogens [11]. These tissue-resident and monocyte-derived phagocytes are among the first immune cells to encounter invading microbes, where they function as professional phagocytes and immunological decision-makers that shape the magnitude and character of the inflammatory response 9, 11, 12. Upon recognizing microbial ligands via pattern recognition receptors, macrophages undergo polarization, metabolic reprogramming, and transcriptional activation to promote pathogen clearance [12]. Several studies indicate that *KP* can engage both NOD-like receptor family pyrin domain-containing 3 (NLRP3) and NOD-like receptor family CARD domain-containing 4 (NLRC4) inflammasomes to trigger caspase-1- and caspase-11-mediated pyroptosis, while simultaneously activating receptor-interacting protein kinase 3 (RIPK3)-dependent necroptosis in immune cells, thereby exacerbating host tissue injury and impairing bacterial clearance 9, 13, 14. *KP* also manipulates macrophage metabolism and polarization to evade immune clearance, thus promoting the formation of immunosuppressive or dysfunctional subsets 15, 16. Among the regulatory mechanisms controlling macrophage responses, cytosolic sensors are essential for detecting intracellular danger signals and linking mitochondrial stress or nucleic acid sensing to inflammatory gene expression and cell death pathways [17]. Understanding how these cytosolic pathways modulate macrophage activation is critical for uncovering novel mechanisms of immune dysregulation in severe bacterial infections.

Z-DNA binding protein 1 (ZBP1), also known as DNA-dependent activator of interferon (IFN)-regulatory factor, is a cytosolic nucleic acid sensor that plays a pivotal role in innate immune responses during microbial infection 18, 19, 20, 21. Upon recognizing pathogen-derived or self-derived nucleic acids in the Z-form (Z-DNA or Z-RNA) via its Zα domains, ZBP1 initiates signaling cascades leading to the production of type I interferon (IFN-I), activation of nuclear factor κB (NF-κB), and assembly of multiprotein complexes called PANoptosomes 18, 19. ZBP1 activation is tightly regulated at both transcriptional and post-translational levels, and its expression is inducible by IFN-I, IFN-II, lipopolysaccharide (LPS), and cytosolic mitochondrial DNA [18]. In bacterial infections, accumulating evidence indicates that ZBP1 participates in host responses to Gram-negative bacteria 22, 23, 24, 25. Li *et al*. [22] showed that IFN-I-induced ZBP1 activation contributes to pyroptotic and necroptotic cell death during endotoxemia, thus linking ZBP1 to IFN-driven inflammatory injury. Muendlein *et al.* 23, 24 further demonstrated that ZBP1 cooperates with TIR-domain-containing adaptor-inducing interferon-β (TRIF) and receptor-interacting serine/threonine-protein kinase 1 (RIPK1) to amplify Toll-like receptor (TLR)3/4-mediated cytokine production and endotoxic shock, underscoring its role in LPS-triggered inflammatory amplification. Consistently, Pearson *et al.* [25] reported that enteropathogenic *Escherichia coli* (*E. coli*) uses the cysteine protease effector EspL to cleave ZBP1 and other RIP homotypic interaction motif (RHIM)-containing proteins, thereby blocking necroptosis and inflammation. Together, these studies suggest that ZBP1 functions as a critical RHIM-dependent hub in Gram-negative bacterial infection, balancing antimicrobial defense and immunopathology.

This study aimed to elucidate the molecular mechanism by which macrophage-derived ZBP1 drives dysregulated inflammation during *KP*-induced sepsis. Specifically, we sought to determine how IFN-I-dependent activation of ZBP1 regulates STAT3 signaling and suppresses the expression of Legumain (LGMN), a key mediator of macrophage anti-inflammatory polarization and tissue repair. We further explored how the ZBP1-STAT3-LGMN signaling axis contributes to immune imbalance, barrier disruption, and multi-organ dysfunction, and evaluated whether restoring LGMN activity could mitigate *KP*-induced sepsis.

## 2 Methods

### 2.1 Human blood samples and human peripheral blood mononuclear cells isolation

We prospectively collected blood samples from 17 patients with sepsis and 6 healthy controls (HC) at Jinling Hospital between June 30, 2024, and December 31, 2024. The criteria are provided in **Additional file 1: Methods**. Blood samples were collected 1 d after admission to the surgical intensive care unit. Before collecting blood samples, informed consent was obtained from all human participants. Human peripheral blood mononuclear cells (PBMCs) were isolated by density gradient centrifugation. PBMCs from all 17 sepsis patients were used for mRNA expression analysis, and PBMCs from 3 of these patients were additionally used for protein expression detection. Briefly, 4 ml of Lymphoprep™ (07801; STEMCELL Technologies, Canada) was added to a 15 ml SepMate™ tube (85415; STEMCELL Technologies, Canada). Diluted 2 ml of blood with 2 ml of phosphate buffer saline (PBS), and the diluted blood was layered on top of the Lymphoprep™. The mixture was then centrifuged at 800× *g* for 20 min at room temperature with the brake off. The upper plasma and PBMC layers were collected and further centrifuged at 800× *g* for 3 min at room temperature for subsequent analysis.

All experiments were performed in accordance with government policies and the Declarations of Helsinki and Istanbul. The collection of human blood samples was approved by the Institutional Review Board Ethics Committee at Jinling Hospital (2024DZKY-001-01).

### 2.2 Animals and treatments

A total of 443 mice (6−8 weeks old, on a C57BL/6 background) were used in the experiments, of which 218 wild-type (WT), 51 *Zbp1* knockout (*Zbp1*^*-/-*^), 44 *Ripk3* knockout (*Ripk3*^*-/-*^), 10 interferon alpha/beta receptor 1 knockout (*Ifnar1*^*-/-*^), 37 mixed lineage kinase domain-like protein knockout (*Mlkl*^*-/-*^), 10 stimulator of interferon genes knockout (*Sting*^*-/-*^), 22 myeloid-specific *Zbp1* knockout (*Zbp1*^*fl/fl*^*Lyz2-Cre*^*+/-*^), 22 *Zbp1*^*fl/fl*^*Lyz2-Cre*^*-/-*^, and 29 myeloid-specific *Lgmn* knockout (*Lgmn*^*fl/fl*^*Lyz2-Cre*^*+/-*^, *Lgmn*^*CKO*^) male mice were used. WT, *Ripk3*^*-/-*^ and *Ifnar1*^*-/-*^ mice were purchased from the Model Animals Research Center of Nanjing University (China). Z*bp1*^*-/-*^ mice were gifts from Dr. Rui Gui (Army Medical University, China). *Mlkl*^*-/-*^ mice were gifts from Dr. Jiahuai Han (Xiamen University, China). *Sting*^*-/-*^ mice were purchased from Gempharmatech Co., Ltd. *Zbp1*^*fl/ fl*^*Lyz2-Cre*^*+/-*^ mice were a gift from Dr. Lina Kang (Nanjing University, China). Their littermate *Zbp1*^*fl/fl*^*Lyz2-Cre*^*-/-*^ controls were generated in our animal facility by intercrossing *Zbp1*^*fl/fl*^*Lyz2-Cre*^*+/-*^ mice. *Lgmn*^*CKO*^ mice were purchased from the Shanghai Model Organisms Center.

WT, *Zbp1*^*-/-*^, *Ripk3*^*-/-*^, and *Mlkl*^*-/-*^ Mice were randomly assigned to undergo cecal ligation and puncture (CLP) modeling, and WT, *Zbp1*^*-/-*^, *Ripk3*^*-/-*^, *Mlkl*^*-/-*^, *Ifnar1*^*-/-*^, *Sting*^*-/-*^, *Zbp1*^*fl/fl*^*Lyz2-Cre*^*+/-*^, *Zbp1*^*fl/fl*^*Lyz2-Cre*^*-/-*^ and *Lgmn*^*CKO*^ mice were randomly assigned to intraperitoneal injection with *KP* for infection, with observation lasting up to 14 d. The specific procedures for animal modeling methods are described in **Additional file 1: Methods**. All the mice were housed under specific pathogen-free, ventilated, and thermostatic conditions with a 12-hour light/dark cycle at 24 °C. Food intake was measured over one week using racks in a regular cage that were weighed every week. All animal experiments were carried out in compliance with ethical regulations and were approved by the Institutional Animal Care and Use Committee of Jinling Hospital (DZYISKTB20240317001).

### 2.3 Bacteria preparation

Under sterile conditions, sterile tweezers were used to pick up magnetic bead-bound bacteria and roll them onto a blood agar plate, followed by incubation at 37 °C. The day before performing *in vivo* or *in vitro* experiments, a single bacterial colony was selected from the blood agar plate and cultured overnight in Luria-Bertani (LB) broth with continuous shaking until reaching the exponential growth phase. The bacteria were then washed three times with sterile PBS to remove bacterial mucus and other secretions. The bacterial suspension was quantified and diluted in PBS to an OD600 of 1 [5×10⁸ colony-forming units (CFU)/ml]. In this study, the NTUH-K2044 and ATCC-700603 strains were used for experiments.

### 2.4 *In vivo* bacterial burden assay

To evaluate the bacterial burden in major organs following *KP* infection, mice were sacrificed at the indicated time points, and the blood, intestines, liver, and lungs were collected under sterile conditions. The organs were rinsed gently with sterile PBS to remove surface contaminants and then minced into small pieces using sterile scissors and tweezers. The tissues were homogenized in sterile PBS (1 ml per 100 mg tissue) using a pre-chilled glass homogenizer. The homogenates were serially diluted 10-fold in sterile PBS (10^–1^ to 10^–6^). Aliquots (10 μl) of each dilution were spread onto sterile square Petri dishes (100 mm×15 mm, PS, DNase/RNase-free) containing LB agar using a sterile glass spreader. After allowing the inoculum to absorb for 5–10 min, plates were inverted and incubated at 37 °C for 12 h. CFUs were counted on plates containing 30–300 colonies, and bacterial loads were expressed as log_10_ (CFU/g tissue) or log_10_ (CFU/ml blood). Each measurement was performed in triplicate, and all results were confirmed across at least three independent biological replicates.

### 2.5 Protein extraction and Western blotting

Proteins were extracted from tissues or cells using RIPA lysis buffer (P0013B; Beyotime, China) supplemented with protease (HY-K0010; Med Chem Express, USA) and phosphatase inhibitors (HY-K0021; Med Chem Express, USA). After centrifugation, the supernatants were collected, mixed with SDS loading buffer, and boiled. Proteins were separated by SDS-PAGE, transferred to PVDF membranes (Millipore, USA), blocked with 5% milk, and incubated with primary antibodies overnight at 4 °C, followed by secondary antibodies (7074 or 7076; Cell Signaling Technology, USA) for 1 h at room temperature. Blots were visualized using a TANON 5200 chemiluminescence imaging system (TANON, China), and band intensities were quantified with ImageJ.

### 2.6 Plasmids and transfection

Transfection of cells was performed using Lipofectamine^TM^ 3000 Transfection Reagent (L3000150; Invitrogen, USA) or the Neon Transfection System (Thermo Fisher Scientific, USA) according to the manufacturer’s instructions, and the transfection efficiency was validated by qPCR. The endotoxin-free plasmid pCMV3-GFP-Puro-c6×His-*Lgmn* was purchased from Corues Biotechnology (China). The endotoxin-free plasmids ZBP1-Flag, ZBP1-ΔZα-Flag, ZBP1-ΔZα1-Flag, ZBP1-ΔZα2-Flag, ZBP1-ΔRHIMA-Flag, ZBP1-ΔRHIMB-Flag, ZBP1-ΔC-Flag, and transcription factor A, mitochondrial (TFAM)-Flag were purchased from General Biosystems (China).

### 2.7 AAV9-mediated macrophage-specific overexpression of Legumain

To achieve macrophage-specific overexpression of LGMN *in vivo*, an adeno-associated virus 9 (AAV9) vector carrying the murine *Lgmn-*3Fla*g* sequence under the control of the macrophage-specific *F4/80* promoter (VA063-AAV9-F4/80-MCS-3Flag-T2A-ZsGreen) was employed (General Biol, China). The construct was verified by restriction digestion, sequencing, and endotoxin testing according to the manufacturer’s quality-control report.

C57BL/6 mice (6–8 weeks old) were injected via the tail vein with 1×10^11^ plaque-forming units (PFU) of AAV9-F4/80-*Lgmn* in sterile PBS. Control animals received an equivalent dose of AAV9-F4/80-ZsGreen. The *F4/80* promoter ensures selective expression of *Lgmn* in F4/80-positive macrophages throughout the body, enabling systemic macrophage-targeted gene delivery.

All mice were monitored daily for signs of distress or adverse reactions after viral administration and used for subsequent infection or analysis at 2 weeks post-injection.

Other experimental procedures are described in detail in **Additional file 1: Methods**.

### 2.8 Statistical analysis

All statistical analyses were performed using GraphPad Prism 9. Data distribution was assessed for normality prior to statistical testing. For comparisons between two groups, a two-tailed unpaired Student’s *t*-test was used for normally distributed data, while the Mann-Whitney *U* test was applied for non-normally distributed data. For comparisons among multiple groups, one-way or two-way ANOVA followed by appropriate post hoc multiple-comparison tests (Bonferroni or Tukey, as specified in the figure legends) was used when data met parametric assumptions; otherwise, the Kruskal-Wallis test was applied. Data are presented as mean±SD, unless otherwise indicated. *P*<0.05 indicates statistical significance.

## 3 Results

### 3.1 ZBP1 activation in macrophages during *KP*- or CLP-induced sepsis

**Fig. 1:**
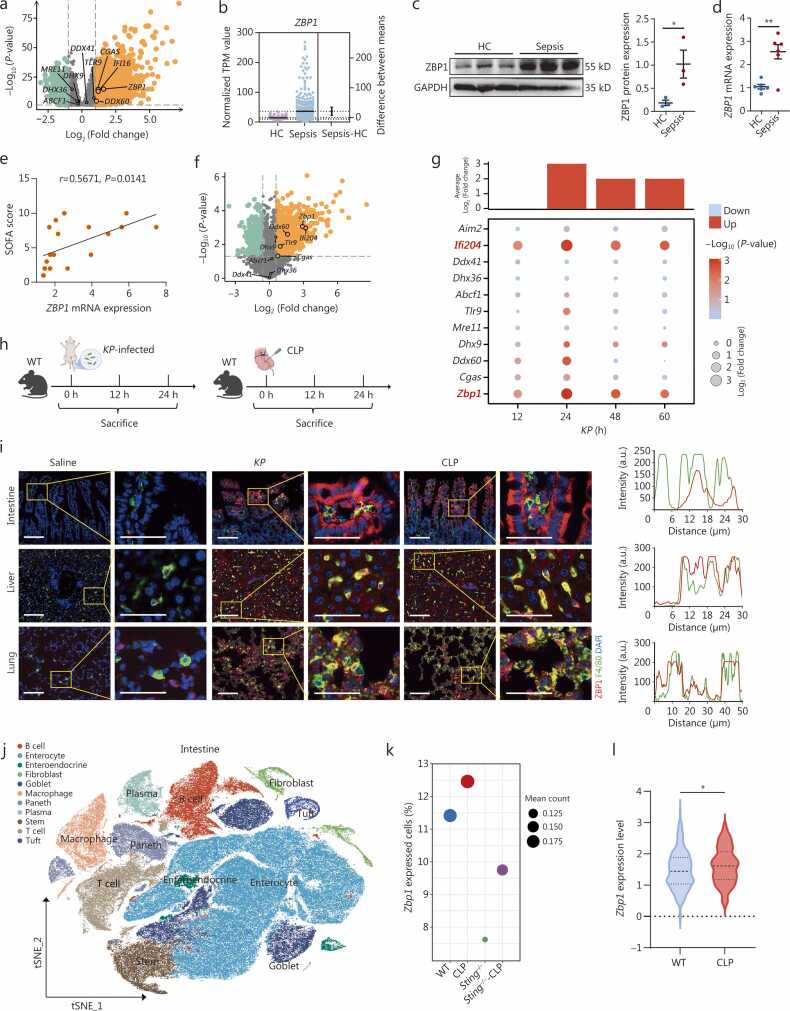
ZBP1 activation in macrophages during *KP* infection in mice and sepsis patients. **a** Volcano plots displaying differentially expressed genes (DEGs) in peripheral blood from sepsis patients compared with healthy controls (HC) in dataset GSE185263. **b** Comparison of *ZBP1* expression levels between sepsis patients and HC. **c** Western blotting analysis of ZBP1 expression in peripheral blood mononuclear cells (PBMCs). **d** RT-qPCR analysis of *ZBP1* expression in PBMCs. **e** Correlation analysis between *ZBP1* mRNA expression and SOFA scores (*n*=17). Correlation analyses were performed using Pearson’s correlation coefficient. **f** Volcano plots displaying DEGs in lungs of *KP*-infected mice (24 h vs. 0 h) in dataset GSE199546. **g** Heatmap of differential gene expression in *KP*-infected mice in dataset GSE199546, comparing lung tissues at 12, 24, 48, and 60 h after *KP* infection with 0 h. **h** Schematic illustration of the *in vivo* experimental timelines. WT mice were subjected to either intraperitoneal *KP* infection (left) or CLP surgery (right) at 0 h. **i** Immunofluorescence (IF) staining of ZBP1 and F4/80 at 24 h in *KP* infection and CLP mice (*n*=3). For the intestine, scale bar=100 μm. For the liver and lung, scale bar=50 μm. **j-l** Single-cell RNA sequencing analysis of *Zbp1* expression in intestinal macrophages from different groups of mice (*n*=3). t-SNE visualization of single-cell transcriptomes (**j**). Ratio of *Zbp1-*expressing cells (**k**). Comparison of *Zbp1* expression levels in cells from the WT and CLP groups (**l**). Data are shown as mean±SD. Statistical significance was assessed using the Mann-Whitney *U* test (**b, l**) and the unpaired two-tailed Student’s *t*-test (**c, d**). *P<0.05, **P<0.01. CGAS. Cyclic GMP-AMP synthase; IFI16. Interferon-γ-inducible protein 16; ZBP1. Z-DNA binding protein 1; DDX60. Dead box polypeptide 60; TLR9. Toll-like receptor 9; DDX41. Dead box polypeptide 41; DHX9. DEXH-box helicase 9; MRE11. Meiotic recombination 11 homolog A; DHX36. DEXH-box helicase 36; ABCF1. ATP-binding cassette subfamily F member 1; HC. Healthy control; GAPDH. Glyceraldehyde-3-phosphate dehydrogenase; SOFA. Sequential Organ Failure Assessment; IFI204. Interferon-activable protein 204; AIM2. Absent in Melanoma 2; *KP*. *Klebsiella pneumoniae*; CLP. Cecal ligation and puncture; t-SNE. t-distributed stochastic neighbor embedding; WT. Wild type; STING. Stimulator of interferon genes. DAPI. 4’,6-diamidino-2-phenylindole.

To first assess the clinical relevance of ZBP1 in sepsis, we analyzed peripheral blood RNA-sequencing data from 348 patients with sepsis and 44 healthy controls (HC) in dataset GSE185263 [26]. Notably, *ZBP1* exhibited robust and consistent upregulation among DNA sensors in sepsis (Fig. 1**a, b; Additional file 1:** Fig. S1a). We further isolated PBMCs from sepsis and confirmed higher ZBP1 protein levels by Western blotting and higher *ZBP1* mRNA expression by qRT-PCR than PBMCs from HC (Fig. 1**c, d**). In addition, *ZBP1* mRNA expression positively correlated with the Sequential Organ Failure Assessment (SOFA) score, supporting its clinical association with disease severity (Fig. 1**e**).

To characterize the dynamic changes in pattern recognition receptors and nucleic acid sensors during *KP* infection, we analyzed lung transcriptomes from *KP*-infected mice at 0, 12, 24, 48, and 60 h post-infection using dataset GSE199546 27, 28, 29. Among the examined DNA sensors, *Zbp1* and *Ifi204* showed the most pronounced and sustained upregulation during *KP* infection (Fig. 1**f, g**). Multiple innate immune receptors were also induced, including C-type lectin receptors (CLR; *Clec4e*, *Fcer1g*, and *Clec7a*), the NOD-like receptor (NLR; *Nlrp3*), TLRs (*Tlr7*, *Tlr6*, *Tlr4*, and *Tlr2*), and the RIG-I-like receptor (RLR; *Ifih1*) (**Additional file 1:** Fig. S1b), highlighting a broad activation of innate immune sensing pathways in response to *KP*. Moreover, the transcriptomic profiles of lungs from *KP*-infected mice and peripheral blood from sepsis revealed similar immune response patterns, suggesting that *KP infection* triggers sepsis-like hyperactivation of the immune system (**Additional file 1:** Fig. S1c**, d**).

To further validate these findings *in vivo*, we established two sepsis models, including an intraperitoneal *KP* infection model and a CLP-induced polymicrobial sepsis model (Fig. 1**h**). To model severe *KP* infection, we compared two standard strains (ATCC-700603 and NTUH-K2044). Compared with ATCC-700603, infection with the NTUH-K2044 strain caused marked mortality in mice (**Additional file 1:** Fig. S2a). After NTUH-K2044 infection, the spleen, liver, and lung weights increased from 6 to 24 h, and the spleen became darkened over time (**Additional file 1:** Fig. S2b). Histological analysis further showed that NTUH-K2044 induced more severe tissue injury in the intestine, liver, and lung than ATCC-700603 **(Additional file 1:** Fig. S2c**)**. In terms of ZBP1 induction, ATCC-700603 did not induce significant upregulation of ZBP1, while NTUH-K2044 triggered a much more pronounced upregulation of ZBP1 expression **(Additional file 1:** Figs. S2d**, e** and **S3a)**. Macrophages infected with NTUH-K2044 showed results with marked activation of the IFN-I inflammatory (JAK1-STAT1) signaling pathway **(Additional file 1:** Fig. S2f**, g)**. Therefore, we selected the NTUH-K2044 strain for subsequent experiments (abbreviated as *KP*).

Consistent with the transcriptomic results, ZBP1 expression was elevated in the intestine, liver, and lung of mice at 24 h in *KP*-infected and CLP mice (**Additional file 1:** Fig. S3a**-d**). Tissue immunofluorescence (IF) further showed colocalization of ZBP1 with macrophages (F4/80 positive) (Fig. 1**i**). In parallel, analysis of our single-cell RNA-sequencing data from mice intestinal tissues showed that the proportion of *Zbp1*-positive macrophages was increased in the CLP group, whereas *Sting* deficiency reduced the proportion of *Zbp1*-positive macrophages **(**Fig. 1**j, k; Additional file 1:** Fig. S3e**)**, and that *Zbp1* expression levels of macrophages were also higher in CLP mice **(**Fig. 1**l)**.

Collectively, these human and animal data consistently demonstrate that ZBP1 is markedly upregulated in macrophages during *KP*-induced sepsis and is associated with disease severity.

### 3.2 ZBP1 deletion ameliorates sepsis and organ injury during *KP-* or CLP-induced sepsis

**Fig. 2:**
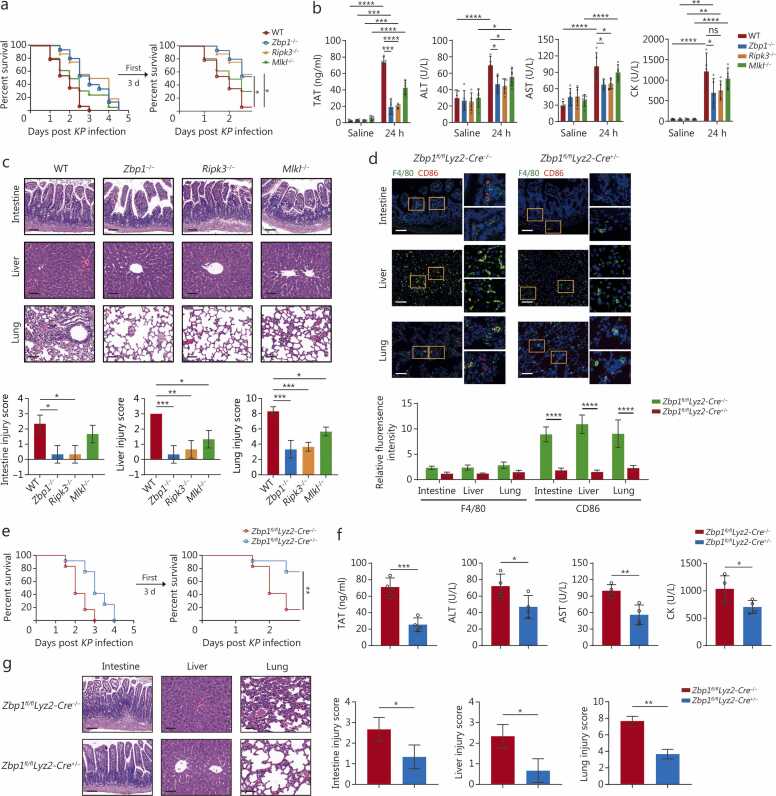
*Zbp1* deficiency ameliorates *KP*-induced sepsis and organ injury. **a** Kaplan-Meier survival curve of *KP*-infected mice with different genotypes (1000 CFU) (WT, *n*=32; *Zbp1*^*-/-*^, *n*=15; *Ripk3*^*-/-*^, *n*=16; *Mlkl*^*-/-*^, *n*=16). Survival differences were analyzed using the log-rank (Mantel-Cox) test. **b** Serum levels of TAT, ALT, AST, and CK at 24 h *KP*-infection (4000 CFU) (*n*=5). **c** H&E staining and histopathological assessment of intestinal, liver, and lung tissues at 24 h *KP*-infected (4000 CFU) mice (*n*=3). For the intestine, scale bar=100 μm. For the liver and lung, scale bar=50 μm. **d** Immunofluorescence (IF) analysis of intestine, liver, and lung tissues from *Zbp1*^*fl/fl*^*Lyz2-Cre*^*-/-*^ and *Zbp1*^*fl/fl*^*Lyz2-Cre*^*+/-*^ mice at 24 h post *KP* infection (4000 CFU) showing macrophage marker F4/80 (green) and M1 activation marker CD86 (red). Quantification of relative fluorescence intensity (*n*=3). For the intestine, scale bar=100 μm. For the liver and lung, scale bar=50 μm. Enlarged insets highlight the colocalization of F4/80 and CD86 signals. **e** Survival analysis of *Zbp1*^*fl/fl*^*Lyz2-Cre*^*-/-*^ and *Zbp1*^*fl/fl*^*Lyz2-Cre*^*+/-*^ mice following *KP* infection (4000 CFU) (*n*=12). Survival differences were analyzed using the log-rank (Mantel-Cox) test. **f** Serum levels of TAT, ALT, AST, and CK at 24 h *KP*-infection (4000 CFU) in *Zbp1*^*fl/fl*^*Lyz2-Cre*^*-/-*^ and *Zbp1*^*fl/fl*^*Lyz2-Cre*^*+/-*^ mice (*n*=4). **g** H&E staining sections of intestine, liver, and lung tissues at 24 h after *KP* infection (4000 CFU) in *Zbp1*^*fl/fl*^*Lyz2-Cre*^*-/-*^ and *Zbp1*^*fl/fl*^*Lyz2-Cre*^*+/-*^ mice (*n*=3). For the intestine, scale bar=100 μm. For the liver and lung, scale bar=50 μm. Data are shown as mean±SD. Statistical significance was assessed using the two-way ANOVA followed by Tukey’s multiple comparisons test (**b**), the Kruskal-Wallis test followed by Dunn’s multiple comparisons test (**c**), and the unpaired two-tailed Student’s *t*-test (**d-g**). *P<0.05, **P<0.01, ***P<0.001, ****P<0.0001, ns non-significant. *KP*. *Klebsiella pneumoniae*; WT. Wild type; ZBP1. Z-DNA binding protein 1; RIPK3. Receptor-interacting protein kinase 3; MLKL. Mixed lineage kinase domain-like protein; TAT. Thrombin-antithrombin complex; ALT. Alanine transaminase; AST. Aspartate aminotransferase; CK. Creatine kinase.

To determine whether ZBP1 activation in macrophages drives the pathogenesis of *KP*-induced sepsis, we investigated the disease severity in *Zbp1*^*-/-*^, *Ripk3*^*-/-*^, and *Mlkl*^*-/-*^ mice. *KP* infection was lethal for mice of all genotypes; however, *Zbp1-* and *Ripk3-*deficience significantly delayed the progression of fatality in infected mice **(**Fig. 2**a**). Kaplan-Meier survival analysis showed that *Zbp1*^*-/-*^ and *Ripk3*^*-/-*^ mice showed significantly improved survival compared with that of WT mice after CLP, while the survival of *Mlkl*^*-/-*^ mice was intermediate **(Additional file 1:** Fig. S4a**)**. Accordingly, thrombin-antithrombin complexes (TAT), alanine aminotransferase (ALT), aspartate aminotransferase (AST), and creatine kinase (CK) levels at 24 h post-sepsis modeling were lower in *Zbp1*^*-/-*^ mice compared with WT mice, while TAT, ALT, and AST levels were also reduced in *Ripk3*^*-/-*^ mice (Fig. 2**b; Additional file 1:** Fig. S4b). Furthermore, histopathological analysis of intestinal, liver, and lung tissue sections revealed that *Zbp1*^*-/-*^ and *Ripk3*^*-/-*^mice exhibited lower injury scores compared with WT mice in the sepsis model. The saline groups showed essentially no detectable tissue injury (Fig. 2**c; Additional file 1:** Fig. S4c**, d**).

Given these findings, we employed *Zbp1*^*fl/fl*^*Lyz2-Cre*^*+/-*^ to further determine the contribution of macrophage ZBP1 to *KP* infection-induced inflammation and organ injury. IF staining revealed extensive accumulation of F4/80^+^CD86^+^ macrophages in the intestine, liver, and lungs of *Zbp1*^*fl/fl*^*Lyz2-Cre*^*-/-*^ mice after *KP* infection, whereas this inflammatory infiltration was markedly reduced in *Zbp1*^*fl/fl*^*Lyz2-Cre*^*+/-*^ mice, and the saline groups showed essentially no detectable tissue injury (Fig. 2**d**; **Additional file 1:** Fig. S4e). Consistently, following *KP* infection, *Zbp1*^*fl/fl*^*Lyz2-Cre*^*+/-*^ mice exhibited prolonged survival compared with *Zbp1*^*fl/fl*^*Lyz2-Cre*^*-/-*^ mice, and serum TAT, ALT, AST, and CK levels were reduced (Fig. 2**e, f**). Histopathological examination further revealed reduced tissue injury in the intestine, liver, and lungs of myeloid ZBP1-deficient mice. The saline groups showed no difference (Fig. 2**g; Additional file 1:** Fig. S4f).

To evaluate whether bacterial burden differences contributed to the observed phenotypes, we quantified bacterial loads in the blood, spleen, liver, and lung of infected mice. No significant differences in organ weights or *KP* colony counts were detected between mice of different genotypes, indicating that bacterial burden did not account for the improved survival and reduced organ injury observed in *Zbp1*^*-/-*^ or *Zbp1*^*fl/fl*^*Lyz2-Cre*^*+/-*^ mice (**Additional file 1:** Fig. S5a**-d**). Macrophages from mice of different genotypes also exhibited comparable phagocytic activity toward *KP* infection (**Additional file 1:** Fig. S5e).

Together, these results demonstrate that myeloid *Zbp1* deletion attenuates macrophage activation, systemic inflammation, and organ injury during *KP*-induced sepsis.

### 3.3 Type I interferon signaling and mitochondrial stress are critical for ZBP1 activation during *KP* infection

**Fig. 3:**
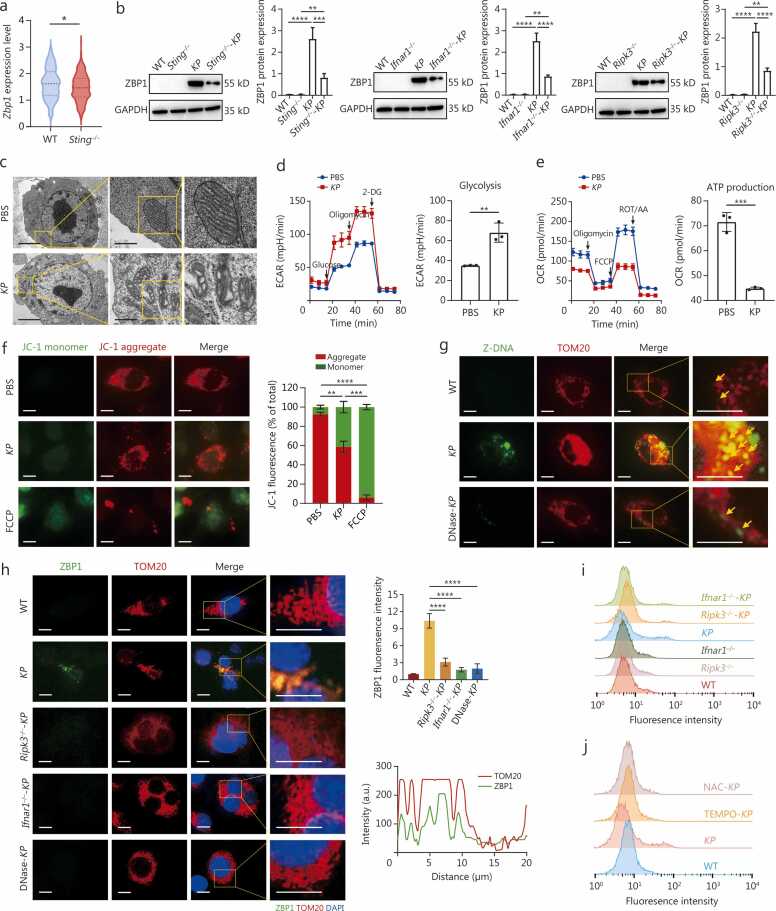
Type I interferon-mediated mitochondrial damage is essential for ZBP1 activation during *Klebsiella pneumoniae* (*KP*) infection. **a** Single-cell RNA sequencing analysis comparing *Zbp1* expression in intestinal macrophages of *Sting*^*-/-*^ and WT mice following CLP. **b** Western blotting analysis of ZBP1 protein expression in BMDMs isolated from WT, *Sting*^*-/-*^, *Ifnar1*^*-/-*^, and *Ripk3*^*-/-*^ mice following *KP* infection (MOI=50:1, 12 h, *n*=3). **c** Transmission electron microscopy of mitochondrial damage in BMDMs post-infection (MOI=50:1, 6 h). For the original images, scale bar=2 μm. For the enlarged images, scale bar=500 nm. **d** Seahorse glycolysis stress test in BMDMs treated with PBS or infected with *KP* (MOI=50:1, 6 h, *n*=3). The real-time extracellular acidification rate (ECAR) (left). The quantification of glycolytic capacity (right). **e** Seahorse mitochondrial stress test in BMDMs treated with PBS or infected with *KP* (MOI=50:1, 6 h) (*n*=3). The real-time oxygen consumption rate (OCR) (left). Quantification of OCR-derived ATP production (right). **f** Assessment of mitochondrial membrane potential in BMDMs by JC-1 staining. Representative fluorescence images showing JC-1 monomers (green) and JC-1 aggregates (red) in control, *KP*-infected, and FCCP-treated macrophages (MOI=50:1, 6 h, *n*=5). Scale bar=5 μm. **g** Visualization of Z-DNA accumulation in BMDMs following *KP* infection. Representative immunofluorescence images showing Z-DNA (green) and the mitochondrial marker TOM20 (red) in WT, *KP*-infected and DNase-treated *KP*-infected BMDMs (MOI=50:1, 6 h, *n*=5). Scale bars=5 μm. **h** Representative immunofluorescence images showing ZBP1 (green), the mitochondrial marker TOM20 (red), and nuclei (DAPI, blue) in control, *KP*, *Ripk3*^*-/-*^-*KP*, *Ifnar1*^*-/-*^-*KP* and DNase- *KP* BMDMs (MOI=50:1, 6 h) (*n*=3). Scale bar=5 μm. **i** Representative flow cytometry histograms of Mito SOX fluorescence in WT, *Ripk3*^*-/-*^, and *Ifnar1*^*-/-*^ BMDMs under control conditions and after *KP* infection (MOI=50:1, 6 h) (*n*=3). **j** Representative flow cytometry histograms of Mito SOX fluorescence in control and *KP*-infected macrophages pre-treated with NAC (100 μmol/L, 1 h) or TEMPO (10 μmol/L, 1 h) (MOI=50:1, 6 h) (*n*=3). Data are presented as mean±SD. Statistical significance was assessed using the Mann-Whitney *U* test (**a**), the Kruskal-Wallis test followed by Dunn’s multiple comparisons test (**b**), the unpaired two-tailed Student’s *t*-test (**d, e**), and the one-way ANOVA followed by Dunnett’s multiple comparisons test (**f, h**). *P<0.05, **P<0.01, ***P<0.001, ****P<0.0001. ZBP1. Z-DNA binding protein 1; WT. Wild type; STING. Stimulator of interferon genes; GAPDH. Glyceraldehyde-3-phosphate dehydrogenase; *KP*. *Klebsiella pneumoniae*; IFNAR1. Interferon alpha/ beta receptor subunit 1; RIPK3. Receptor-interacting serine/threonine-protein kinase 3; PBS. Phosphate-buffered saline; ECAR. Extracellular acidification rate; OCR. Oxygen consumption rate; ATP. Adenosine triphosphate; TOM20. Translocase of outer mitochondrial membrane 20; DAPI. 4’,6-diamidino-2-phenylindole; DNase. Deoxyribonuclease; NAC. N-acetylcysteine; TEMPO. 2,2,6,6-tetramethylpiperidin-1-oxyl.

We next explored the mechanism driving ZBP1 upregulation in macrophages during *KP* infection. *Zbp1* is an IFN-stimulated gene, and our results indicate that the IFN-I signaling pathway is activated in both *KP* infection and in PBMCs from sepsis (**Additional file 1:** Fig. S6a). Single-cell RNA sequencing analysis further revealed that deletion of *Sting* reduced *Zbp1* expression in macrophages (Fig. 3**a**). We then stimulated BMDMs from WT, *Sting*^*-/-*^, *Ifnar1*^*-/-*^, and *Ripk3*^*-/-*^ mice with *KP* to assess ZBP1 induction. The results showed that loss of key molecules in the IFN signaling pathway markedly impaired ZBP1 activation (Fig. 3**b**). The mRNA expression of *Zbp1*, *Ifit1*, *Ifit3*, *Irf4*, *Irf5*, and *Irf7* also confirmed that IFN-stimulated gene expression was significantly lower in *Ripk3*^-/-^ and *Ifnar1*^-/-^ BMDMs post-infection, accompanied by suppression of the canonical IFN-mediated JAK1-STAT1 signaling pathway (**Additional file 1:** Fig. S6b**, c**). At the tissue level, ZBP1 expression was reduced in the intestines of *Sting*^*-/-*^, *Ripk3*^*-/-*^, and *Ifnar1*^*-/-*^ mice at 24 h after *KP* infection (**Additional file 1:** Fig. S6d**, e**). Collectively, these findings indicate that Sting-mediated IFN-I signaling is essential for ZBP1 activation in macrophages during *KP* infection.

ZBP1 is a sensor for mitochondria-derived Z-DNA in multiple experimental models, where it triggers downstream immune responses 18, 30, 31, 32. In parallel, *KP* infection induces regulated mitochondrial stress, manifested by mitochondrial fragmentation, ER-mitochondria Ca²⁺ overload, and cytosolic release of mitochondrial DNA; these events collectively amplify innate immune signaling [13]. We observed that *KP* infection triggered mitochondrial ultrastructural alterations in BMDMs, including disrupted cristae organization, vacuole formation, and a decreased frequency of intact cristae (Fig. 3**c**). Seahorse metabolic flux analysis showed that *KP*-infected cells had a higher extracellular acidification rate (ECAR) and lower oxygen consumption rate (OCR)-derived ATP production than the control group (PBS) (Fig. 3**d, e**). The mitochondrial membrane potential of *KP*-infected BMDMs was also reduced (Fig. 3**f**). These findings were consistent with an increased B-cell lymphoma 2 (BCL-2)-associated X protein (BAX)/BCL2 ratio and reduced TFAM expression at 12 and 24 h after *KP* infection, indicating mitochondrial genomic instability (**Additional file 1:** Fig. S7a). TFAM is essential for maintaining mitochondrial DNA packaging and genome stability; its reduction is widely regarded as a marker of mitochondrial genome instability and has been shown to promote ZBP1 activation [33]. *Tfam* knockdown increased ZBP1 protein expression, whereas TFAM overexpression reduced *KP*-induced ZBP1 upregulation (**Additional file 1:** Fig. S7b**, c**). Further examination of Z-DNA levels revealed increased accumulation of Z-DNA in *KP*-infected BMDMs, which colocalized with mitochondria (Fig. 3**g**). In *KP*-infected BMDMs, upregulated ZBP1 also colocalized with mitochondria, and the accumulation of both ZBP1 and Z-DNA was effectively abolished by DNase treatment, suggesting that this process depends on Z-mtDNA production (Fig. 3**g, h**). Consistently, MitoSOX staining showed reduced mitochondrial reactive oxygen species (ROS) signals in *Ripk3*^*-/-*^ and *Ifnar1*^*-/-*^ BMDMs compared with WT groups after *KP* infection (Fig. 3**i**). Increased mitochondrial ROS can promote mitochondrial damage, and we therefore asked whether this process contributes to ZBP1 activation. After treatment with the antioxidants 2,2,6,6-tetramethylpiperidin-1-oxyl (TEMPO) or N-acetyl cysteine (NAC), the *KP* infection-induced increase in mitochondrial ROS was reversed (Fig. 3**j**). TEMPO and NAC treatment also effectively reduced ZBP1 activation in *KP*-infected BMDMs (**Additional file 1:** Fig. S7d).

Together, these results indicate that both IFN-I signaling and mitochondrial stress are important requirements for ZBP1 activation in macrophages during *KP* infection.

### 3.4 *Zbp1* deletion facilitates tissue remodeling and ameliorates intestinal barrier injury during *KP* infection

**Fig. 4:**
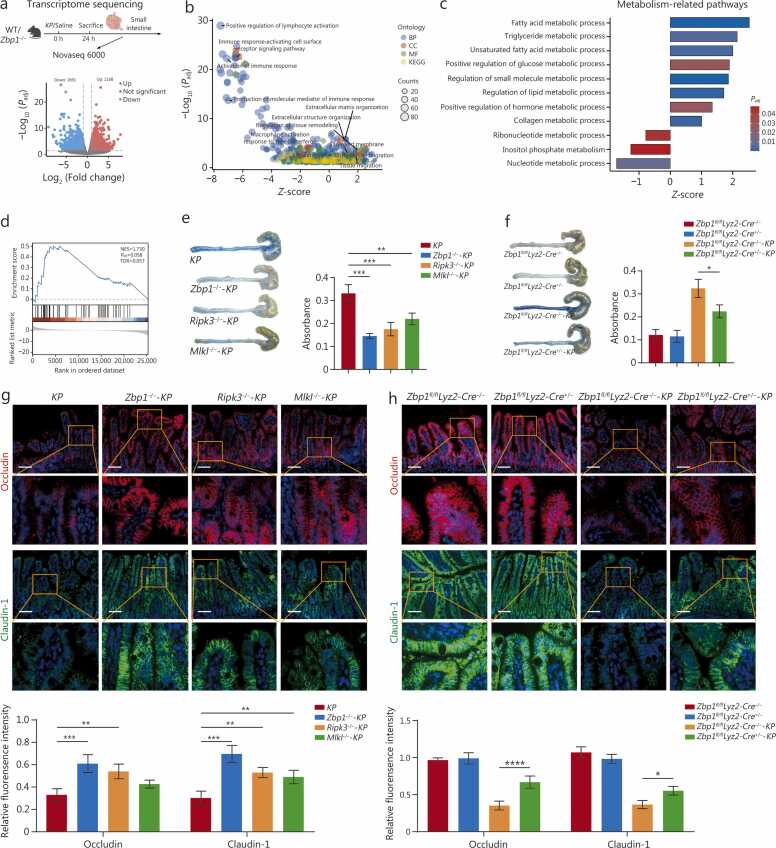
*Zbp1* deletion facilitates tissue remodeling and ameliorates intestinal barrier injury during *Klebsiella pneumoniae* (*KP*) infection. **a** Schematic diagram of tissue collection timeline following *KP* infection (4000 CFU). Volcano plot showing differentially expressed genes (DEGs) (WT-*KP* vs. *Zbp1*^*-/-*^-*KP* mice) (*n*=3). **b** KEGG and GO enrichment analysis of DEGs based on combined log_2_ fold change between WT-*KP* and *Zbp1*^*-/-*^-*KP* mice. **c** Reactome pathway enrichment analysis of metabolic processes among DEGs between WT-*KP* and *Zbp1*^*-/-*^-*KP* mice. **d** GSEA comparing intestinal transcriptomes of WT-*KP* and *Zbp1*^*-/-*^-*KP* mice after *KP* infection revealed a significant enrichment of genes associated with regulation of tissue remodeling. **e** Representative images of Evans blue-stained intestines from WT, *Zbp1*^*-/-*^, *Ripk3*^*-/-*^, and *Mlkl*^*-/-*^ mice at 24 h post-*KP* infection (4000 CFU). Quantitative analysis of absorbance per tissue weight (mg) indicates decreased vascular leakage in *Zbp1*^*-/-*^ mice compared with WT, while *Ripk3*^*-/-*^ and *Mlkl*^*-/-*^ mice showed similar trends (*n*=3). **f** Representative Evans blue staining of intestines from *Zbp1*^*fl/fl*^*Lyz2-Cre*^*-/-*^ and *Zbp1*^*fl/fl*^*Lyz2-Cre*^*+/-*^ mice following saline or *KP* infection (4000 CFU) (*n*=3). **g** Representative immunofluorescence images of intestinal sections stained for Occludin (red) and Claudin-1 (green) from *KP*, *Zbp1*^*-/-*^, *Ripk3*^*-/-*^ and *Mlkl*^*-/-*^ mice at 24 h post-infection (4000 CFU). Scale bar=100 μm. Quantification of fluorescence intensity shows enhanced tight junction preservation in *Zbp1*^*-/-*^ mice compared with *KP* groups (*n*=3). **h** Immunofluorescence analysis of Occludin (red) and Claudin-1 (green) in intestines from *Zbp1*^*fl/fl*^*Lyz2-Cre*^*-/-*^ and *Zbp1*^*fl/fl*^*Lyz2-Cre*^*+/-*^ mice after *KP* infection (4000 CFU) (*n*=3). Scale bar=100 μm. Data are shown as mean±SD. Statistical significance was assessed using the Kruskal-Wallis test followed by Dunn’s multiple comparisons test (**e-h**). *P<0.05, **P<0.01, ***P<0.001, ****P<0.0001. ZBP1. Z-DNA binding protein 1; WT. Wild type; *KP*. *Klebsiella pneumoniae*; BP. Biological process; CC. Cellular component; MF. Molecular function; KEGG. Kyoto encyclopedia of genes and genomes; RIPK3. Receptor-interacting protein kinase 3; MLKL. Mixed lineage kinase domain-like protein.

To gain deeper insight into the regulatory mechanisms of ZBP1 during *KP* infection, we performed high-throughput transcriptomic profiling of small intestinal tissues from infected mice (Fig. 4**a**). In total, 2859 differentially expressed genes (DEGs), including 1168 upregulated genes and 1691 downregulated genes were identified (WT-*KP* vs. *Zbp1*^*-/-*^-*KP*) (Fig. 4**a**). Next, we performed Kyoto Encyclopedia of Genes and Genomes (KEGG) and Gene Ontology (GO) pathway enrichment analyses based on the combined log_2_ (fold change) in genotype and treatment. The results showed that genes associated with immune response, lymphocyte activation, macrophage activation, and IFN-I response were significantly downregulated, whereas genes related to tissue remodeling, angiogenesis, and extracellular matrix reorganization were significantly upregulated (Fig. 4**b**). Specifically, changes in metabolism-related pathways suggest that *Zbp1* deletion shifts metabolism toward lipid utilization, reduces inflammation-associated nucleotide metabolism, and promotes tissue repair mechanisms (Fig. 4**c; Additional file 1:** Fig. S8a). Notably, macrophage lipid synthesis contributes to the production of regeneration-related factors [34].

GSEA further showed enrichment of the tissue remodeling signature in *Zbp1*^*-/-*^-*KP* mice compared with WT-*KP* mice (Fig. 4**d**). We further explored the effects of *Zbp1* deficiency on the intestinal epithelium with compromised barrier function in *KP*-infected mice. Following *KP* challenge, WT mice showed marked Evans blue extravasation, whereas *Zbp1*^*-/-*^, *Ripk3*^*-/-*^, and *Mlkl*^*-/-*^ mice exhibited significantly lower intestine-normalized absorbance, indicating reduced barrier leakage (Fig. 4**e**). Consistently, *Zbp1*^*fl/fl*^*Lyz2-Cre*^*+/-*^ mice also decreased Evans blue accumulation compared with the *Zbp1*^*fl/fl*^*Lyz2-Cre*^*-/-*^ group in *KP*-infection, whereas saline groups showed no difference between genotypes (Fig. 4**f**). IF staining of tight junction proteins further demonstrated that *KP* infection markedly disrupted intestinal epithelial barrier integrity in WT mice, characterized by reduced Occludin and Claudin-1 expression (Fig. 4**g**). In contrast, *Zbp1*^*-/-*^, *Ripk3*^*-/-*^, and *Mlkl*^*-/-*^ mice exhibited a preserved tight junction structure and significantly higher fluorescence intensity of Occludin and Claudin-1, indicating attenuated epithelial barrier damage (Fig. 4**g**). Similarly, *Zbp1*^*fl/fl*^*Lyz2-Cre*^*+/-*^ mice restored tight junction protein expression in infected mice, as reflected by increased Claudin-1 expression and reduced Occludin loss compared with the saline-treated group (Fig. 4**h**).

Thus, both systemic and myeloid-specific loss of *Zbp1* facilitates tissue remodeling and protects the intestinal barrier against *KP*-induced injury.

### 3.5 *Zbp1* deficiency mitigates macrophage inflammation via Legumain

**Fig. 5:**
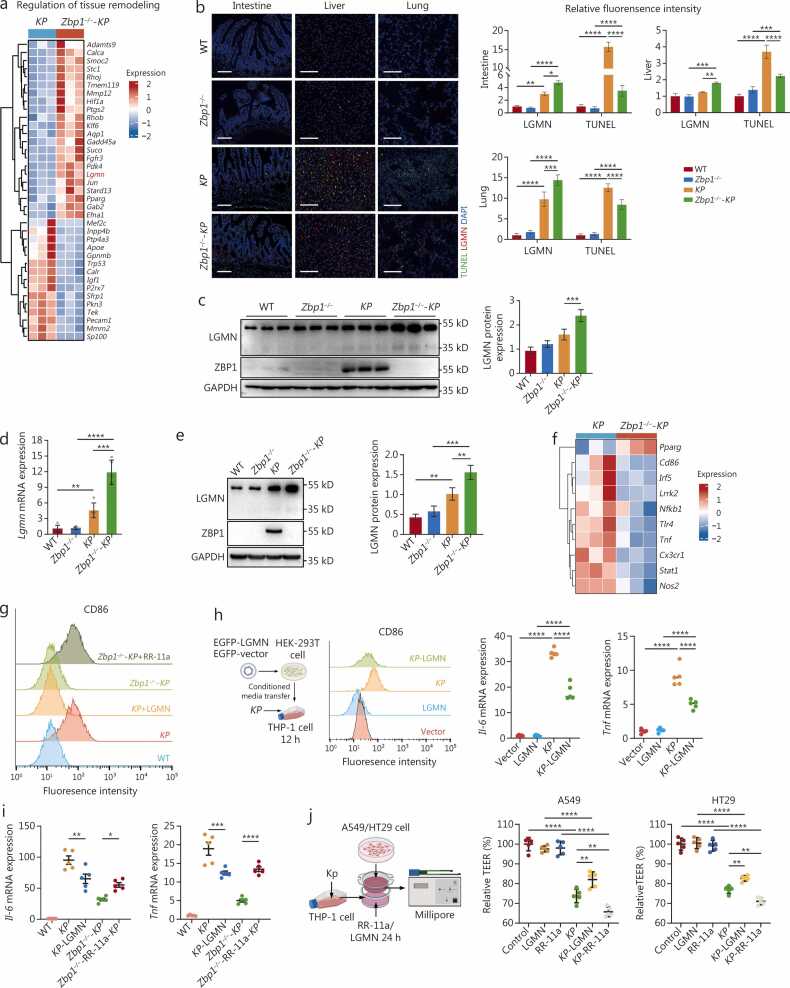
*Zbp1* deficiency mitigates macrophage inflammation via Legumain (LGMN). **a** Heatmap of genes enriched in tissue remodeling-related pathways based on intestinal transcriptomic analysis of *KP*-infected WT and *Zbp1*^*-/-*^ mice. **b** Immunofluorescence staining of intestine, liver, and lung from *KP*-infected mice (4000 CFU, 24 h) to assess LGMN expression and apoptotic cell (TUNEL) distribution (*n*=3). Scale bar=100 μm. **c** Detection of LGMN protein levels in the intestinal tissues of *KP*-infected mice (4000 CFU, 24 h) (*n*=3). **d** Transcriptional levels of *Lgmn* in WT and *Zbp1*^*-/-*^ BMDMs following *KP* infection (MOI=50:1, 6 h) (*n*=3). **e** Protein levels of LGMN in WT and *Zbp1*^*-/-*^ BMDMs following *KP* infection (MOI=50:1, 6 h) (*n*=3). **f** Heatmap of differentially expressed genes enriched in the macrophage M1 polarization pathway between *Zbp1*^*-/-*^-*KP* and *KP* groups. **g** Flow cytometric analysis of CD86 expression in macrophages under control conditions and following *KP* infection (MOI=50:1, 12 h) (*n*=5). Representative histograms show CD86 expression in WT and *Zbp1*^*-/-*^ macrophages. WT macrophages were treated with recombinant LMGN (50 ng/ml), and *Zbp1*^*-/-*^ macrophages were treated with the LGMN inhibitor RR-11a (50 nmol/L). **h** Schematic diagram of conditioned medium transfer from LGMN-overexpressing HEK-293T cells to THP-1 cells, followed by *KP* infection (MOI=50:1, 12 h). Flow cytometry analysis of CD86 expression in THP-1 cells (*n*=5) (left). Relative mRNA expression of inflammatory genes in THP-1 cells co-cultured with supernatants from EGFP-LGMN-transfected HEK-293T cells compared to Vector controls (*n*=5) (right). **i** Relative mRNA expression of inflammatory genes in WT and *Zbp1*^*-/-*^ BMDMs infected with *KP* (MOI=50:1, 12 h) with or without LMGN (50 ng/ml) and RR-11a (50 nmol/L) pretreatment (*n*=5) for 1 h. **j** Schematic diagram of the co-culture experimental design. THP-1 cells were infected with *KP* (MOI=50:1) for 12 h prior to co-culture. RR-11a (50 nmol/L) and LMGN (50 ng/ml) were added 1 h prior to co-culture. TEER measurements were recorded starting at 0 h of co-culture. HT29 and A549 cells had formed confluent monolayers prior to co-culture. Data showing disruption of epithelial barrier integrity in A549 and HT29 monolayers following co-culture with infected THP-1 cells. The effects of LMGN (50 ng/ml) and RR-11a (50 nmol/L) were assessed in independent treatment groups (*n*=5). Data are shown as mean±SD. Statistical significance was assessed using the Kruskal-Wallis test followed by Dunn’s multiple comparisons test (**b, c, e**), the Two-way ANOVA followed by Tukey’s multiple comparisons test (**d, h, j**), and the one-way ANOVA followed by Dunnett’s multiple comparisons test (**i**). *P<0.05, **P<0.01, ***P<0.001, ****P<0.0001. ZBP1. Z-DNA binding protein 1; Adamts9. A disintegrin and metalloproteinase with thrombospondin motifs 9; Calca. Calcitonin/calcitonin-related polypeptide alpha; Smoc2. SPARC-related modular calcium-binding protein 2; Stc1. Stanniocalcin 1; Rhoj. Ras homolog family member J; Tmem119. Transmembrane protein 119; Mmp12. Matrix metallopeptidase 12; Hif1a. Hypoxia-inducible factor 1 subunit alpha; Ptgs2. Prostaglandin-endoperoxide synthase 2; Rhob. Ras homolog family member B; Klf6. Kruppel-like factor 6; Aqp1. Aquaporin 1; Gadd45a. Growth arrest and DNA damage-inducible alpha; Suco. SUN domain-containing ossification factor; Fgfr3. Fibroblast growth factor receptor 3; Pak4. p21-activated kinase 4; Jun. Jun proto-oncogene, AP-1 transcription factor subunit; Stard13. StAR-related lipid transfer domain containing 13; Pparg. Peroxisome proliferator-activated receptor gamma; Gab2. GRB2-associated binding protein 2; Efna1. Ephrin A1; Mef2c. Myocyte enhancer factor 2 C; Inpp4b. Inositol polyphosphate-4-phosphatase type II B; Ptp4a3. Protein tyrosine phosphatase 4A3; Apoe. Apolipoprotein E; Gpnmb. Glycoprotein nonmetastatic melanoma protein B; Trp53. Transformation related protein 53; Calr. Calreticulin; Igf1. Insulin-like growth factor 1; P2rx7. Purinergic receptor P2X 7; Sfrp1. Secreted frizzled-related protein 1; Pkn3. Protein kinase N3; Tek. TEK receptor tyrosine kinase; Pecam1. Platelet and endothelial cell adhesion molecule 1; Mmrn2. Multimerin 2; Sp100. SP100 nuclear antigen; WT. Wild type; *KP. Klebsiella pneumoniae*; LGMN. Legumain; TUNEL. Terminal deoxynucleotidyl transferase dUTP nick end labeling; DAPI. 4',6-diamidino-2-phenylindole; GAPDH. Glyceraldehyde-3-phosphate dehydrogenase; Irf5. Interferon regulatory factor 5; Lrrk2. Leucine-rich repeat kinase 2; Nfkb1. Nuclear factor kappa B subunit 1; Tlr4. Toll-like receptor 4; Tnf. Tumor necrosis factor; Cx3cr1. C-X3-C motif chemokine receptor 1; Stat1. Signal transducer and activator of transcription 1; Nos2. Nitric oxide synthase 2; IL-6. Interleukin-6; TEER. Transepithelial electrical resistance; EGFP. Enhanced green fluorescent protein.

Having observed the protective effects of macrophage ZBP1 deficiency, we further analyzed the transcriptomic profiles of WT and *Zbp1*^-/-^ mice to delineate the relevant downstream mechanisms. Analysis of upregulated tissue remodeling pathways revealed 22 significantly upregulated genes, including *Lgmn* (Fig. 5**a**). Macrophage LGMN is involved in promoting apoptotic cell clearance and facilitating tissue repair, and also serves as a marker of M2 polarization, which counteracts the proinflammatory state 35, 36, 37. This prompted us to investigate the role of LGMN during *KP* infection. IF staining of the mice intestine, liver, and lung tissues showed an increase of LMGN in *Zbp1*^*-/-*^-*KP* groups, accompanied by a reduction in apoptotic cells as indicated by TUNEL staining (Fig. 5**b**). Co-localization of LGMN with TUNEL-positive cells suggested that LGMN may be involved in the clearance of apoptotic cells. In intestinal tissues, LGMN protein expression was higher in *Zbp1*^*-/-*^-*KP* than in WT mice after *KP* infection(Fig. 5**c**). In BMDMs, *KP* infection increased both LGMN mRNA and protein expression, with higher levels in *Zbp1*^*-/-*^ cells than in WT cells (Fig. 5**d, e**). LGMN expression was also upregulated in *Sting*^*-/-*^ mice following *KP* infection, indicating that this pathway is dependent on IFN-I signaling (**Additional file 1:** Fig. S8b).

To further elucidate the *in vivo* role of LGMN in modulating inflammatory responses and influencing survival outcomes in mice, RR-11a was administered intraperitoneally at 6 h before and 12 h after sepsis induction to inhibit endogenous LGMN activity. Consistent with our findings, RR-11a treatment significantly reduced the survival of *Zbp1*^*-/-*^ mice (**Additional file 1:** Fig. S8c). Furthermore, inhibiting LGMN notably exacerbated multi-organ injury in *Zbp1*^*-/-*^ mice (**Additional file 1:** Fig. S8d**, e**), thereby underscoring the potential of LGMN as a therapeutic target during *KP* infection.

We then analyzed the expression of inflammatory macrophage-related genes in the intestines of infected mice, where genes related to M1 macrophage polarization (*Cd86*, *Irf5*, *Lrrk2*, *Nfkb1*, *Tlr4*, *Tnf*, *Cx3cr1*, *Stat1*, and *Nos2*) were significantly downregulated in the *Zbp1*^*-/-*^ group (Fig. 5**f**). To verify whether upregulated LGMN affects the inflammatory state of macrophages, the total macrophage population (F4/80^+^CD11b^+^), including M1 macrophages (F4/80^+^CD11b^+^CD86^+^) and M2 macrophages (F4/80^+^CD11b^+^CD206^+^), from the bone marrow of *Zbp1*^*-/-*^ and WT mice was analyzed using flow cytometry (**Additional file 1:** Fig. S9a). *Zbp1*^*-/-*^-treated with infected with *KP* or LPS combined with IFN-γ showed reduced M1 polarization, as indicated by lower CD86 expression, which was reversed by RR-11a (an irreversible inhibitor of LGMN), while exogenous LMGN reduced CD86 expression in WT macrophages under the same conditions (Fig. 5**g; Additional file 1:** Fig. S9b). Co-culture with the EGFP-LGMN-HEK-293T cells supernatant suppressed M1 polarization in THP-1 cells, and LGMN could decline *Il-6* and *Tnf* mRNA expression in THP-1 cells (Fig. 5**h; Additional file 1:** Fig. S9c). Subsequently, IL-4 stimulation induced M2 polarization, as reflected by an increased proportion of CD206-positive macrophages compared with the control group. This M2 population was further increased in the *Zbp1*^*-/-*^-IL-4 and IL-4+LGMN groups, whereas RR-11a treatment markedly reduced the proportion of M2 macrophages in *Zbp1*^*-/-*^-IL-4+RR-11a cells (**Additional file 1:** Fig. S9d). LMGN also attenuated the expression of proinflammatory genes (*Il-6, Tnf*) in BMDMs following infection, whereas RR-11a treatment increased *Il-6* and *Tnf* mRNA expression in *Zbp1*^*-/-*^ BMDMs relative to the untreated *Zbp1*^*-/-*^ group (Fig. 5**i**).

As we demonstrated that myeloid-specific *Zbp1* deficiency improves epithelial barrier integrity *in vivo*, we established a macrophage-epithelial co-culture model to determine whether this effect depends on macrophage-derived LGMN. A549 and HT29 cells showed reduced transepithelial electrical resistance (TEER) at 12 and 24 h after co-culture with supernatants from *KP*-infected THP-1 cells, consistent with reduced barrier function (**Additional file 1:** Fig. S9e). Exogenous LMGN attenuated the TEER reduction in HT29 and A549 cells exposed to supernatants from *KP*-infected THP-1 cells, whereas RR-11a further enhanced this reduction, suggesting that macrophage-derived LGMN influences epithelial barrier function in neighboring cells (Fig. 5**j**). Further, TEER measurements in A549 and HT29 cells co-cultured with THP-1 and supernatants from HEK-293T cells transfected with EGFP-LGMN were significantly increased at 12 and 24 h compared with the EGFP-vector group, supporting the barrier-stabilizing function of endogenously released LGMN (**Additional file 1:** Fig. S9f).

Together, these data highlight that LGMN plays a pivotal role in regulating the macrophage inflammatory response and may serve as a potential target for controlling inflammation during *KP* infection.

### 3.6 ZBP1 binds with STAT3 to regulate Legumain expression

**Fig. 6:**
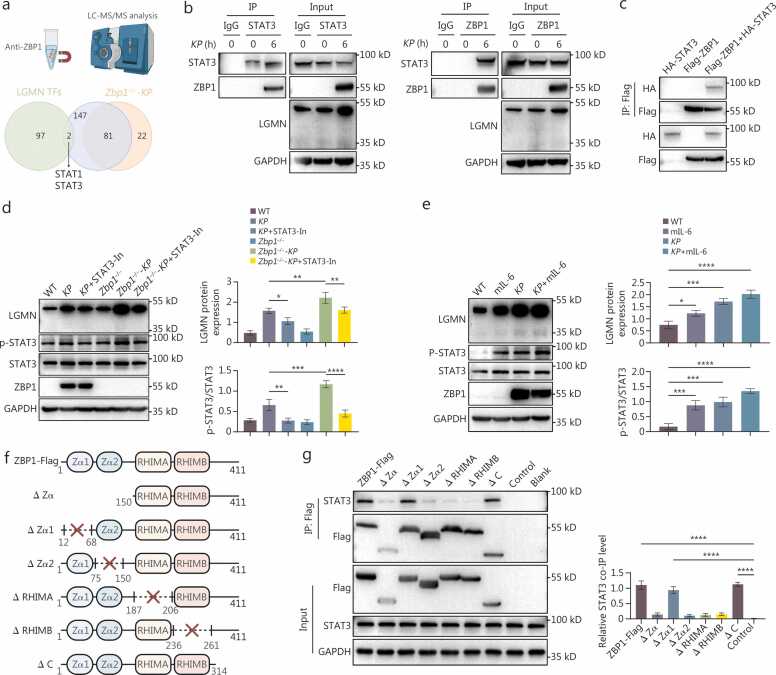
ZBP1 binds to STAT3 to regulate Legumain (LGMN) expression. **a** Schematic diagram of the workflow for immunoprecipitation, mass spectrometry, and candidate protein screening. Proteins from *KP*-infected (MOI=50:1, 3 h) WT and *Zbp1*-deficient BMDMs were subjected to anti-*Zbp1* immunoprecipitation followed by mass spectrometry analysis. **b** Co-immunoprecipitation of ZBP1 and STAT3 in BMDMs infected with *KP* (MOI=50:1, 6 h). **c** Co-immunoprecipitation of HA-STAT3 and ZBP1-Flag following co-transfection in HEK-293T cells. **d** Immunoblot analysis of LGMN, phosphor-Y705-STAT3 (p-STAT3), STAT3, and ZBP1 protein levels in WT and *Zbp1*^*-/-*^ BMDMs following *KP* infection (MOI=50:1, 6 h). STAT3 inhibitor (Stattic, Stat3-In) (2.5 μmol) was used as a Stat3 inhibitor (*n*=3). **e** Immunoblot analysis of LGMN, phosphor-Y705-STAT3 (p-STAT3), STAT3, and ZBP1 protein levels in WT BMDMs following *KP* infection (MOI=50:1, 6 h). IL-6 (30 ng/ml, 6 h) as a Stat3 activator (*n*=3). **f** Schematic representation of mouse ZBP1 truncation constructs. **g** Co-immunoprecipitation of ZBP1-Flag truncation constructs and STAT3 in HEK-293T cells transfected with plasmids expressing different ZBP1 truncations (*n*=3). Data are shown as mean±SD. Statistical significance was assessed using the Kruskal-Wallis test followed by Dunn’s multiple comparisons test (**d-g**). *P<0.05, **P<0.01, ***P<0.001, ****P<0.0001, ns non-significant. LC-MS/MS. Liquid chromatography-tandem mass spectrometry; LGMN. Legumain; TF. Transcription factor; IP. Immunoprecipitation; ZBP1. Z-DNA binding protein 1; WT. Wild type; *KP*. *Klebsiella pneumoniae*; STAT1. Signal transducer and activator of transcription 1; STAT3. Signal transducer and activator of transcription 3; GAPDH. Glyceraldehyde-3-phosphate dehydrogenase; p-STAT3. Phosphor-Y705- STAT3; RHIM. RIP homotypic interaction motif.

Considering the increased LGMN expression observed in *Zbp1*-deficient macrophages upon *KP* infection, we proceeded to explore the regulatory pathways involved. Using the hTF-target prediction database (http://swisstargetprediction.ch/), we identified 99 transcription factors potentially regulating *LGMN* (**Additional file 1:** Table S1). We hypothesized that ZBP1 exerts its function by interacting with one of these transcription factors. To test this, we isolated proteins from *KP* and *Zbp1*^*-/-*^-*KP* BMDMs and performed anti-ZBP1 immunoprecipitation followed by liquid chromatography-tandem mass spectrometry (LC-MS/MS) analysis (Fig. 6**a**). After subtracting the proteins identified in the *Zbp1*^*-/-*^ group, we identified 149 candidate proteins potentially interacting with ZBP1, among which only STAT1 and STAT3 were located upstream of *LGMN* in transcriptional regulation (Fig. 6**a**). Transcriptomic profiling revealed markedly reduced *Stat1* expression in *Zbp1*^*-/-*^-*KP* tissues compared with *KP* tissues in WT mice, prompting to shift our focus toward assessing the transcriptional activity of STAT3 as a potential regulator (Fig. 5**f**). Protein-protein interaction (PPI) network analysis of ZBP1-interacting proteins revealed that both ZBP1 and STAT3 occupy central positions within the regulatory network (**Additional file 1:** Fig. S10a). Mass spectrometry identified the STAT3 peptide sequence VCIDKDSGDVAALRGSR as the highest-confidence ZBP1-binding region, and sequence alignment showed that this binding site is highly conserved among human STAT3 isoforms and across multiple species (**Additional file 1:** Fig. S10b**, c**). Consistently, KEGG and GO enrichment analysis of these ZBP1-binding proteins revealed pathways related to RNA splicing, mRNA processing, and IFN-I production, suggesting their potential roles in transcriptional regulation (**Additional file 1:** Fig. S10d).

Next, we performed protein-protein docking simulation and co-immunoprecipitation to validate the interaction between ZBP1 and STAT3. Endogenous binding of ZBP1 and STAT3 was detected in *KP*-infected BMDMs (Fig. 6**b; Additional file 1:** Fig. S10e). Furthermore, co-transfection of HA-tagged STAT3 and Flag-tagged ZBP1 plasmids in HEK-293T cells confirmed their interaction upon simultaneous expression (Fig. 6**c**). The transcriptional activity of STAT3 depends on its phosphorylation (Y705)-mediated activation, and we next investigated whether ZBP1 binding to STAT3 affects its transcriptional activity and thereby regulates LGMN expression. We found that phosphorylation of STAT3 (Y705) was markedly elevated in *KP*-infected *Zbp1*^*-/-*^ compared with WT in BMDMs. In contrast, treatment with a STAT3 inhibitor (Stattic, Stat3-In) partially reversed the LGMN upregulation (Fig. 6**d**). STAT3 phosphorylation was detectable as early as 1 h after infection. In contrast, the transcriptional induction of *Lgmn* was mainly observed after 3 h, suggesting that STAT3 first translocates to the nucleus to promote *Lgmn* transcription, followed by increased protein expression at 6 h (**Additional file 1:** Fig. S11a). LPS is also sufficient to directly induce ZBP1 and LMGN expression (**Additional file 1:** Fig. S11b). *Stat3* knockdown also reduced infection-induced LMGN expression in macrophages but did not alter ZBP1 induction, confirming that STAT3 positively regulates LMGN production (**Additional file 1:** Fig. S11c). IL-6 is a natural activator of STAT3 and promotes its transcriptional activity [38]. We observed that stimulation with mIL-6 alone significantly elevated LGMN expression, indicating that STAT3 activation partially contributes to the upregulation of LGMN expression (Fig. 6**e**). Notably, pharmacological inhibition of STAT3 markedly disrupted the epithelial barrier integrity of HT29 cells when co-cultured with THP-1 macrophages (**Additional file 1:** Fig. S11d).

ZBP1 contains two major structural domains: the Zα domain and the RHIM [18]. Based on published literature, we generated plasmid constructs encoding domain-deletion mutants of *Zbp1* and transfected them into HEK-293T cells, which endogenously expresses STAT3 but lacks basal ZBP1 expression (Fig. 6**f**). Deletion of Zα2, RHIM-A, or RHIM-B was found to impair the interaction between ZBP1 and STAT3, whereas deletion of Zα1 or the C-terminal region had no apparent effect (Fig. 6**g**). Further, IF staining of intestinal, liver, and lung tissues from *KP*-infected mice revealed a co-expression pattern of ZBP1 and STAT3 (**Additional file 1:** Fig. S11e).

Taken together, our results suggest that *KP* infection-induced upregulation of ZBP1 facilitates its interaction with STAT3, potentially contributing to the transcriptional regulation of LGMN.

### 3.7 ZBP1 inhibits the nuclear translocation of STAT3 to suppress *Lgmn* transcription

**Fig. 7:**
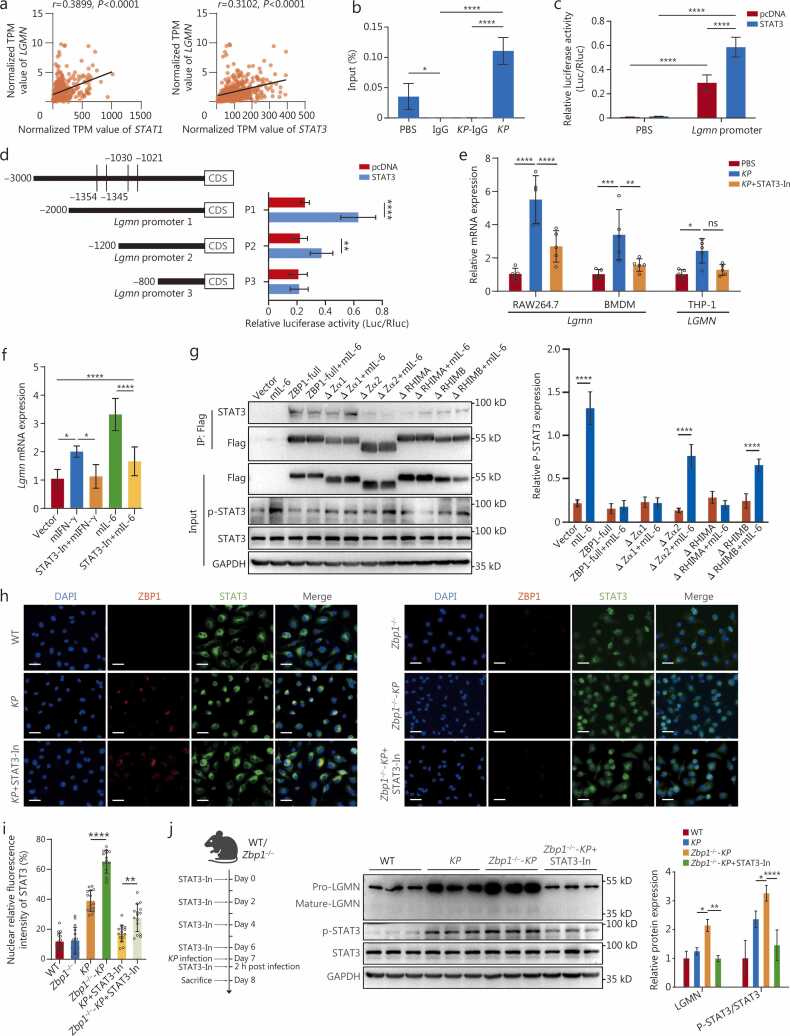
ZBP1 inhibits the nuclear translocation of STAT3 to suppress *Lgmn* transcription. **a** Correlation analysis between *LGMN* expression and *STAT1/STAT3* in peripheral blood from sepsis patients (GSE185263). Correlation analyses were performed using Pearson’s correlation coefficient. **b** Chromatin immunoprecipitation (ChIP) assay demonstrating STAT3 binding to the *Lgmn* promoter in *KP*-infected (MOI=50:1, 6 h) BMDMs (*n*=4). DNA fragments immunoprecipitated with anti-STAT3 antibodies were amplified by PCR, showing a distinct *Lgmn* promoter band in the STAT3-IP sample compared with the IgG group. **c** Dual-luciferase reporter assay performed in HEK-293T cells transfected with either an *LGMN* promoter-driven reporter plasmid or an empty control vector, together with pcDNA or STAT3 expression constructs (*n*=6). **d** Schematic representation of *Lgmn* promoter truncation constructs (P1–P3) containing different upstream regions relative to the transcription start site (–3000 to –800 bp) and their corresponding luciferase activities (*n*=6). **e** RT-qPCR analysis of *Lgmn* transcription levels in three types of monocyte/macrophage cells following *KP* infection (MOI=50:1, 6 h) (*n*=5). STAT3 inhibitor (Stattic, Stat3-In) was used to inhibit STAT3 activation (2.5 μmol/L). **f** BMDMs were stimulated with recombinant murine IFN-γ (20 ng/ml, 6 h) and IL-6 (30 ng/ml, 6 h), and *Lgmn* transcription levels were analyzed (*n*=5). **g** HEK-293T cells were transfected with ZBP1 truncation constructs and treated with recombinant murine IL-6 to activate STAT3 (30 ng/ml, 30 min). The interaction between STAT3 and Flag-tagged ZBP1 and phosphorylation at STAT3 Y705 were assessed (*n*=3). **h, i** Immunofluorescence staining of STAT3 and ZBP1 in BMDMs infected with *KP* infection (MOI=50:1, 6 h). Each group randomly selected 15 cells. Scale bar=50 μm. **j***In vivo* analysis of intestinal tissues from mice treated with intraperitoneal Stat3-In (10 mg/kg) to inhibit STAT3 activation, followed by *KP* infection (4000 CFU, 24 h). Protein levels of LGMN, P-STAT3, total STAT3, and GAPDH were measured (*n*=3). Data are shown as mean±SD. Statistical significance was assessed using the one-way ANOVA followed by Dunnett’s multiple comparisons test (**b, e, f**), the Two-way ANOVA followed by Tukey’s multiple comparisons test (**c, d, g, i**), and the Kruskal-Wallis test followed by Dunn’s multiple comparisons test (**j**). *P<0.05, **P<0.01, ***P<0.001, ****P<0.0001, ns non-significant. LGMN. Legumain; STAT1. Signal transducer and activator of transcription 1; STAT3. Signal transducer and activator of transcription 3; PBS. Phosphate buffered saline; *KP*. *Klebsiella pneumoniae*; DAPI. 4’,6-diamidino-2-phenylindole; IL-6. Interleukin-6; IFN-γ. Interferon-γ; GAPDH. Glyceraldehyde-3-phosphate dehydrogenase; IP. Immunoprecipitation; p-STAT3. phosphor-Y705- STAT3; RHIM. RIP homotypic interaction motif.

Analysis using the JASPAR database to verify the transcriptional regulation of *Lgmn* by STAT3 revealed that the predicted STAT3-binding motifs align with the promoter region of *Lgmn* in both humans and mice, and are evolutionarily conserved across species (**Additional file 1:** Fig. S12a). Transcriptomic analysis of peripheral blood from patients with sepsis revealed a significant positive correlation between *LGMN* expression and both *STAT1* and *STAT3* (Fig. 7**a**). To determine this, we performed chromatin immunoprecipitation (ChIP) using anti-STAT3 antibodies, followed by PCR amplification of the *Lgmn* promoter region. ChIP-qPCR showed minimal enrichment in the PBS-IgG and *KP*-IgG, whereas STAT3 immunoprecipitation enriched the *Lgmn* promoter fragment in both groups, with significantly greater enrichment after *KP* stimulation, confirming that *KP* stimulation enhances STAT3 binding to the *Lgmn* promoter (Fig. 7**b**). To further verify the transcriptional regulation, a luciferase reporter assay was conducted in HEK-293T cells transfected with either the *Lgmn* promoter construct or control plasmid. Luciferase activity remained similarly low in the control reporter group transfected with either pcDNA or STAT3, whereas in the *LGMN* promoter reporter group, STAT3 transfection significantly increased luciferase activity compared with pcDNA (Fig. 7**c**). To map the STAT3-binding region within the *Lgmn* promoter, truncated constructs (P1–P3) spanning –3000 to –800 bp were tested (Fig. 7**d**). The P1 fragment (–2000 to CDS) showed the strongest STAT3-induced luciferase activity, indicating that the key STAT3-responsive sites are located between –2000 and –800 bp. STAT3 overexpression significantly increased *Lgmn* promoter-driven luciferase activity compared with the pcDNA, whereas the control reporter showed no difference. We also performed RT-qPCR analysis in three types of monocytic/macrophage cells: RAW264.7, BMDMs, and THP-1 cells. Treatment with a Stat3-In markedly suppressed the *KP* infection-induced *Lgmn* transcriptional upregulation (Fig. 7**e**). Furthermore, after inducing STAT3 phosphorylation (Y705) with recombinant murine IFN-γ and IL-6, LGMN mRNA and protein levels remained inhibited in the presence of the STAT3-In (Fig. 7**f; Additional file 1:** Fig. S12b). These findings indicate that STAT3 is a key regulatory factor initiating *Lgmn* transcription.

STAT3 phosphorylation at the Y705 residue facilitates its nuclear translocation and transcriptional activity [38]. We hypothesized that the interaction between ZBP1 and STAT3 may inhibit this phosphorylation and nuclear translocation. To test this, HEK-293T cells were transfected with plasmids encoding various ZBP1 truncation constructs, followed by transient activation of STAT3 using recombinant mIL-6 (Fig. 7**g**). ZBP1 fragments lacking Zα2 or RHIMB failed to bind STAT3 and did not affect STAT3 phosphorylation, whereas full-length ZBP1 and the ΔZα1 construct retained STAT3 binding capacity and significantly suppressed STAT3 phosphorylation (Fig. 7**g**). ZBP1 lacking RHIMA failed to bind STAT3 normally, whereas STAT3 phosphorylation was aberrantly activated. IF staining of BMDMs further revealed that *KP* infection increased STAT3 nuclear translocation in *Zbp1*^*-/-*^ BMDMs, an effect abrogated by treatment with a STAT3-In (Fig. 7**h, i**). In contrast, STAT3 in WT cells strongly colocalized with upregulated ZBP1 following *KP* infection or IFN-γ stimulation (Fig. 7**h, i; Additional file 1:** Fig. S12c). Finally, repeated intraperitoneal administration of a STAT3 inhibitor in mice to suppress STAT3 transcriptional activity resulted in the abrogation of intestinal LGMN activation following *KP* infection (Fig. 7**j**).

Together, these findings demonstrate that ZBP1 binds STAT3 endogenously to modulate its nuclear translocation and transcriptional activity.

### 3.8 Macrophage Legumain modulates organ damage and survival during *Klebsiella pneumoniae* infection

**Fig. 8:**
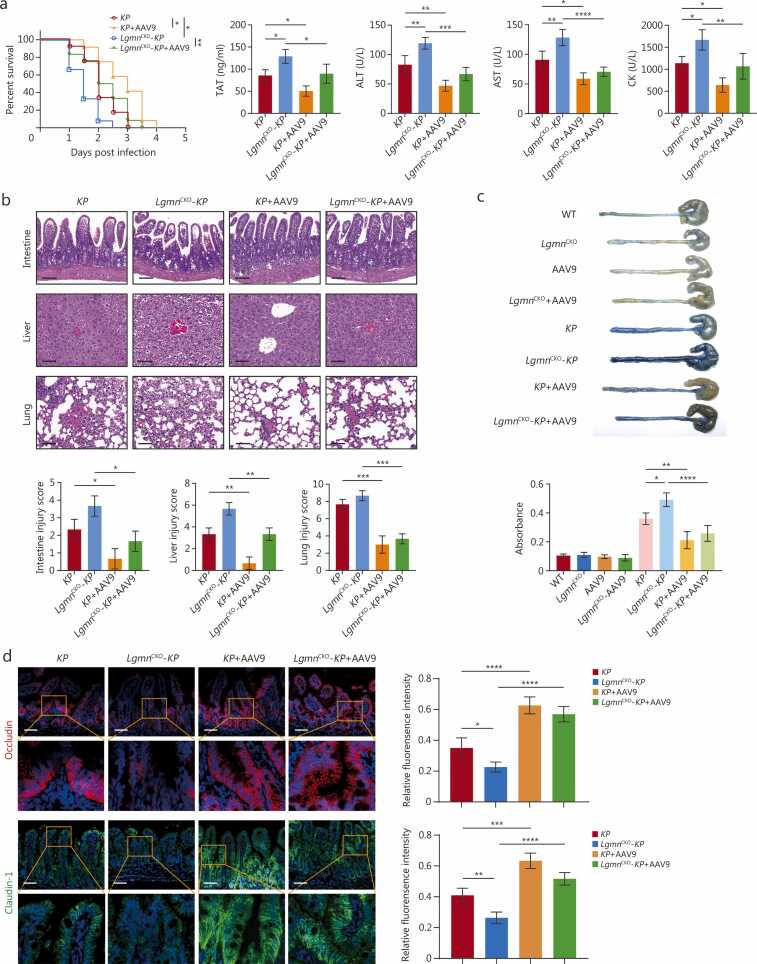
Macrophage Legumain modulation by genetic or pharmacological approaches affects organ damage and survival during *KP* infection. **a** Survival analysis of WT, *Lgmn*^*CKO*^, WT-AAV9 and *Lgmn*^*CKO*^-AAV9 mice following *KP* infection (*n*=12) (left). Survival differences were analyzed using the log-rank (Mantel-Cox) test (right). Serum levels of thrombin-antithrombin complex (TAT), alanine aminotransferase (ALT), aspartate aminotransferase (AST), and creatine kinase (CK) were measured 24 h post infection (4000 CFU) (*n*=4). Data are shown as mean±SD. Statistical significance was assessed using the one-way ANOVA followed by Dunnett’s multiple comparisons test. **b** Representative H&E-stained sections of intestine, liver and lung tissues from WT, *Lgmn*^*CKO*^, WT-AAV9 and *Lgmn*^*CKO*^-AAV9 mice after *KP* infection. For the intestine, scale bar=100 μm. For the liver and lung, scale bar=50 μm. Quantitative scoring (intestine, liver, and lung injury scores) revealed aggravated pathology in *Lgmn*^*CKO*^ mice and marked histological improvement after AAV9-LGMN treatment (*n*=3). Data are shown as mean±SD. **c** Evans blue extravasation assay showing intestinal vascular permeability in saline- or *KP*-treated (4000 CFU, 24 h) mice. Absorbance per tissue weight (mg) quantification indicated increased intestinal leakage in *Lgmn*^*CKO*^ mice, which was significantly reduced following macrophage-specific Legumain overexpression (*n*=3). Data are shown as mean±SD. **d** Representative immunofluorescence images of intestinal tight junction proteins Occludin (red) and Claudin-1 (green) from WT, *Lgmn*^*CKO*^, WT-AAV9 and *Lgmn*^*CKO*^-AAV9 mice at 24 h post *KP* infection (4000 CFU). For the intestine, scale bar=100 μm. For the liver and lung, scale bar=50 μm. Quantitative analysis of fluorescence intensity shows disrupted epithelial junctions in *Lgmn*^*CKO*^ mice, while AAV9-mediated LGMN restoration preserved Occludin and Claudin-1 expression (*n*=3). Data are shown as mean±SD. Statistical significance was assessed using the Kruskal-Wallis test followed by Dunn’s multiple comparisons test (**b, c, d**). *P<0.05, **P<0.01, ***P<0.001, ****P<0.0001. LGMN. Legumain; *KP*. *Klebsiella pneumoniae*; AAV. Adeno-associated virus; WT. Wild type.

We investigated the role of LGMN in the resistance of mice to *KP* infection using *in vivo* experiments. We utilized *Lgmn*^*CKO*^ mice and an AAV9 vector expressing LGMN under the *F4/80* promoter to achieve macrophage-targeted overexpression. Following *KP* infection, *Lgmn*^*CKO*^ mice showed shorter survival and higher serum TAT, ALT, AST, and CK levels than WT mice, AAV9-mediated LGMN overexpression in WT mice reduced these injury-associated indices, and AAV9-mediated LGMN restoration in *Lgmn*^*CKO*^ mice partially reversed the aggravated phenotype, as reflected by prolonged survival and reduced serum marker levels compared with untreated *Lgmn*^*CKO*^ mice (Fig. 8**a**). Histological analysis revealed that intestinal, liver, and lung injury was exacerbated in *Lgmn*^*CKO*^ mice but significantly ameliorated in AAV9-mediated LGMN restoration groups (Fig. 8**b**). Evans blue extravasation assays further demonstrated increased intestinal vascular leakage in *Lgmn*^*CKO*^ mice, which was rescued by AAV9-mediated LGMN restoration treatment (Fig. 8**c**). IF staining showed disrupted epithelial tight junctions with decreased Occludin and Claudin-1 expression in *Lgmn*^*CKO*^ mice, whereas AAV9-mediated LGMN restoration restored epithelial integrity (Fig. 8**d**).

Overall, these findings demonstrate that myeloid LGMN plays a protective role in *KP*-induced sepsis by maintaining epithelial barrier integrity and limiting organ injury and coagulopathy.

## 4 Discussion

**Fig. 9:**
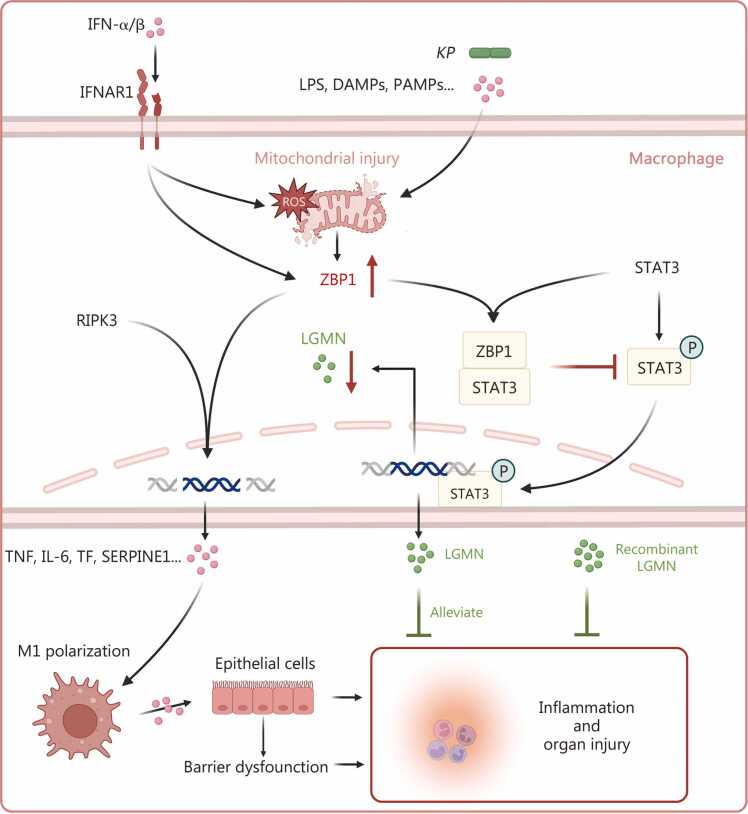
*KP* infection activates type I interferon signaling and induces mitochondrial injury in macrophages, resulting in ZBP1 upregulation. ZBP1 interacts with STAT3, suppressing its phosphorylation and nuclear translocation, thereby inhibiting the transcription of LGMN. Reduced LGMN expression promotes M1 macrophage polarization and upregulates proinflammatory mediators, exacerbating systemic inflammatory disorders. In contrast, recombinant LGMN restores epithelial integrity and alleviates *KP*-induced immunopathology by dampening inflammatory responses. LGMN. Legumain; IFN-α/β. Interferon-α/β; IFNAR1. Interferon alpha/ beta receptor subunit 1; *KP*. *Klebsiella pneumoniae*; LPS. Lipopolysaccharide; DAMPs. Damage-associated molecular patterns; PAMPs. Pathogen-associated molecular patterns; ROS. Reactive oxygen species; ZBP1. Z-DNA binding protein 1; LGMN. Legumain; RIPK3. Receptor-interacting serine/threonine-protein kinase 3; STAT3. Signal transducer and activator of transcription 3; TNF. Tumor necrosis factor; IL-6. Interleukin-6; TF. Tissue factor; SERPINE1. Serpin family E member 1.

In this study, we identify ZBP1 as a key driver of inflammatory injury in *KP*- and CLP-induced sepsis. Genetic deletion of *Zbp1* reduced macrophage inflammation, alleviated organ injury, and improved survival in both *KP*-infection and CLP models, supporting a central role for macrophage-derived ZBP1 in sepsis pathogenesis. Mechanistically, ZBP1 interacted with STAT3 and suppressed its transcriptional activity, thereby reducing LGMN expression. Loss of macrophage-derived LGMN aggravated inflammatory and tissue injury phenotypes, whereas AAV9-mediated LGMN restoration improved disease outcomes. Together, these findings define a novel ZBP1-STAT3-LGMN axis as an important regulatory pathway in bacterial sepsis and highlight LGMN as a potential therapeutic target (Fig. 9).

Compared with the classical strain ATCC-700603, NTUH-K2044 induced a more pronounced activation of ZBP1, which is in line with the more severe inflammatory response and higher virulence typically associated with hv*KP*, as its pathogenic relevance was also supported in the CLP model. Moreover, our analysis of public and in-house clinical data further demonstrated that ZBP1 is upregulated in human sepsis and positively correlated with disease severity, supporting the broader clinical relevance of this pathway.

Among nucleic acid sensors involved in innate immunity, ZBP1 has attracted increasing attention because it functions as an IFN-stimulated gene and can be activated downstream of the cyclic GMP-AMP synthase (cGAS)-stimulator of interferon genes (STING) pathway 39, 40, 41, 42, 43, 44, 45. Previous studies have also shown that excessive IFN-I signaling may impair bacterial clearance during KP infection by promoting macrophage tolerance through IL-10 induction 16, 46, 47. ZBP1 has been reported by Nassour *et al.* [48] to be activated downstream of the cGAS-STING DNA sensing pathway and to require its interaction with TERRA (telomeric-repeat-containing RNA) transcripts, which are produced from dysfunctional telomeres. Here, we screened multiple cytosolic DNA sensors and identified ZBP1 as the only member markedly upregulated during *KP* infection. Its activation required both the IFN-I signaling pathway and mitochondrial stress and was accompanied by the accumulation of Z-form mitochondrial DNA (Z-mtDNA). Previous studies have shown that in Gram-negative infections, ZBP1 can amplify TRIF-RIPK1-dependent inflammatory signaling and cell death, contributing to endotoxin-induced cytokine release and tissue injury 22, 23, 24. Conversely, some bacteria, such as enteropathogenic *E. coli*, degrade ZBP1 through the effector protease EspL to evade necroptosis [25]. Our findings highlight the role of ZBP1 as a critical RHIM-dependent hub that integrates IFN-I signaling and mitochondrial stress.

Both global and *Zbp1*^*fl/fl*^*Lyz2-Cre*^*+/-*^ markedly suppressed systemic inflammation and alleviated multi-organ injury and coagulopathy during *KP* infection, underscoring the dominant role of macrophage-derived ZBP1 in sepsis pathogenesis. Although IFN-I has been reported to promote antimicrobial defense 49, 50, 51, excessive IFN-ZBP1 signaling can drive dysregulated inflammation. In viral infections such as influenza, SARS-CoV-2, and Zika virus, ZBP1 activation triggers RIPK3-dependent necroptosis and cytokine release, contributing to immunopathology 52, 53, 54, 55. Xu *et al.* [6] also observed a therapeutic effect of inflammation suppression following hv*KP* infection. Notably, despite the significant reduction in inflammation and organ injury observed in *Zbp1*^*-/-*^, *Ripk3*^*-/-*^ and *Mlkl*^*-/-*^ mice, bacterial loads in the blood, liver, spleen and lungs remained comparable across genotypes. This indicates that these genes primarily regulate host inflammatory and coagulatory responses rather than influence *KP* replication or clearance, emphasizing that therapeutic benefit arises from controlling excessive inflammation rather than reducing bacterial burden.

Transcriptomic analysis revealed that tissue remodeling and repair pathways were significantly upregulated in the intestines of *Zbp1*^*-/-*^ mice, accompanied by enhanced lipid metabolism and increased Lgmn expression. LGMN is a cysteine protease expressed in the lysosomal system of macrophages [56]. It has been implicated in apoptotic cell clearance and tissue repair in cardiovascular injury models 35, 36. Transcriptional regulation of LGMN involves factors such as HIF1α and STAT3, and this occurs under conditions of lysosomal stress or hypoxia in tumor cells; however, the involvement of LGMN in bacterial infections remains unexplored 57, 58. We observed that ZBP1-deficient macrophages show enhanced phosphorylation of Stat3 (Y705) during *KP* infection, leading to LGMN upregulation and release. Under natural conditions, ZBP1 interacts with STAT3 to suppress its transcriptional activity, thereby limiting LGMN expression. STAT3 plays dual roles in sepsis, with both anti-inflammatory and proinflammatory effects 59, 60. Our findings reveal a new mechanism by which STAT3 promotes anti-inflammatory gene expression in bacteria-infected macrophages, thereby providing an additional layer of insight into the role of STAT3 in bacterial sepsis.

*Lgmn*^*CKO*^ further confirmed the protective role of macrophage-derived LGMN, as its deletion aggravated inflammation, increased fibrin deposition, and disrupted intestinal and vascular barriers, in contrast to the effects of *Zbp1* deficiency or recombinant LGMN treatment. Conversely, macrophage-targeted *Lgmn* overexpression via *F4/80* promoter-driven AAV9 delivery markedly alleviated tissue injury and improved survival after *KP* infection. LGMN also suppressed macrophage inflammation while maintaining epithelial and endothelial integrity; however, these effects were abolished by the inhibitor RR-11a. Wang *et al.* [37] and He *et al.* [61] also reported the anti-inflammatory role of LGMN. Mechanistically, LGMN limits M1 polarization and modulates TRAF6-NF-κB and JAK1/STAT1 signaling to restrain excessive inflammation. Collectively, these findings demonstrate that macrophage-derived LGMN modulates the inflammation induced by bacterial infection and represents a potential therapeutic target.

This study has several limitations. First, although our data indicate that hypervirulent and classical *KP* strains share a core ZBP1-related regulatory pathway, we did not systematically compare a broader panel of hypervirulent, classical, and clinically diverse KP isolates. Moreover, hypervirulent isolates may harbor additional virulence determinants, such as rmpA/rmpA2, iucABCD-iutA, efflux systems, and siderophore biosynthesis genes, that could influence the magnitude and kinetics of ZBP1 activation. Therefore, the specific contribution of individual strain-dependent virulence factors to this mechanism remains to be defined and should be addressed in future studies by directly comparing diverse KP isolates and screening relevant virulence determinants. Second, although our data support the importance of both IFN-I signaling and mitochondrial stress in ZBP1 activation, the precise mechanisms through which IFNAR1 and RIPK3 regulate mitochondrial homeostasis remain unclear, and we cannot conclude from the present study that IFN-I signaling directly causes mitochondrial damage. Third, while our findings support a ZBP1-STAT3-LGMN regulatory axis, we did not perform experiments using STAT3-deficient cell lines or animal models, and such studies will be necessary for more rigorous validation of this pathway. Fourth, although we demonstrated protective effects of ZBP1 deficiency in the intestine, liver, and lung, we did not systematically evaluate other sepsis-vulnerable organs such as the kidney, heart, and brain. Finally, we did not directly test whether exogenous LGMN protein supplementation could confer therapeutic benefit *in vivo*. Because LGMN is synthesized as an inactive precursor and requires intracellular endolysosomal processing for maturation, recombinant protein administration may not faithfully recapitulate the spatiotemporal regulation and functional state of endogenous macrophage-derived LGMN; moreover, systemic delivery would be expected to face substantial tissue-access barriers. Future studies should therefore focus on organ-level validation, genetic dissection of the STAT3 axis, identification of *KP* virulence factors engaging this pathway, and development of engineered LGMN delivery strategies with improved translational feasibility.

## 5 Conclusions

In summary, our results highlight the complex role of ZBP1 during *KP* infection, where it drives inflammation and tissue damage by activating IFN-I signaling and inducing proinflammatory pathways. Therefore, targeting ZBP1 or its downstream effectors, such as LGMN, may provide a promising therapeutic approach for modulating the immune responses in *KP*-induced sepsis and potentially improving clinical outcomes.

## Abbreviations

ALT Alanine aminotransferase

AST Aspartate aminotransferase

BMDM Bone marrow-derived macrophage

CLP Cecal ligation and puncture

CFU Colony-forming unit

CK Creatine kinase

CLSM Confocal laser scanning microscopy

DAMP Damage-associated molecular pattern

DEG Differentially expressed gene

DMEMDulbecco’s Modified Eagle Medium

DMSO Dimethyl sulfoxide

ELISA Enzyme-linked immunosorbent assay

hv*KP* Hypervirulent *KP*

IFN Interferon

IFNAR1Interferon alpha/beta receptor 1

ISG Interferon-stimulated gene

JAK Janus kinase

*KP Klebsiella pneumonia*

LGMN Legumain

LPS Lipopolysaccharide

MLKL Mixed lineage kinase domain-like protein

MOI Multiplicity of infection

NF-κB Nuclear factor κB

NLRP3 NOD-like receptor family pyrin domain-containing 3

PAI-1 Plasminogen activator inhibitor-1

PBMC Peripheral blood mononuclear cell

PBS Phosphate buffer saline

PFA Paraformaldehyde

RHIM RIP homotypic interaction motif

RIPK Receptor-interacting protein kinase

ROS Reactive oxygen species

SOFA Sequential Organ Failure Assessment

STAT Signal transducer and activator of transcription

STING Stimulator of interferon genes

TAT Thrombin-antithrombin complex

TEER Transepithelial electrical resistance

TF Tissue factor

TFAM Transcription factor A, mitochondrial

TUNEL Terminal deoxynucleotidyl transferase dUTP nick end labeling

WT Wild type

ZBP1 Z-DNA binding protein 1

## Ethics approval and consent to participate

All clinical investigations were conducted according to the principles expressed in the Declaration of Helsinki. The collection of human blood samples was approved by the Institutional Review Board Ethics Committee at Jinling Hospital (2024DZKY-001-01). All patients or their legal surrogates provided written informed consent. All animal experiments were carried out in compliance with ethical regulations and were approved by the Institutional Animal Care and Use Committee of Jinling Hospital (DZYISKTB20240317001).

## Funding

This work was supported by the National Natural Science Foundation of China (82272237, 82430083), Jiangsu Provincial Distinguished Young Scholar Program (BK20250108), and Jiangsu Provincial Medical Innovation Center (CXZX202217).

## Data Availability

The datasets used or analyzed during the current study are available from the corresponding author upon reasonable request.

## Footnotes

Appendix A Supplementary data associated with this article can be found in the online version at doi:10.1016/j.mmr.2026.100059.
