# Supplementary material for: ZBP1 activated by mitochondrial damage binds to STAT3 to suppress Legumain production and exacerbate macrophage inflammation in Klebsiella pneumoniae-induced sepsis
Source: Mil Med Res. 2026 Jul 30;13(1):100059. doi: 10.1016/j.mmr.2026.100059 (PMC13452355; doi:10.1016/j.mmr.2026.100059)
Supplement: Supplementary file 1 [file mmc1.pdf]

Methods

Sepsis diagnosis and clinical cohort selection

Sepsis was diagnosed according to the Sepsis 3.0 criteria, defined as life-threatening organ dysfunction caused by a dysregulated host response to infection, with an increase in the Sequential Organ Failure Assessment (SOFA) score of ≥2 points. For the clinical cohort analyzed in this study (excluding public datasets), only patients with culture-confirmed *Klebsiella pneumoniae* (*KP*) infection were included. Patients were excluded if they had a history of autoimmune diseases, malignant tumors, hematological disorders, or chronic inflammatory diseases, or if they had received immunosuppressive therapy, systemic corticosteroids, or chemotherapy prior to admission.

Public dataset acquisition and bioinformatic analysis

GSE185263 and GSE199546 datasets were obtained from the Gene Expression Omnibus (GEO) database. The human sepsis dataset GSE185263, which contains peripheral blood leukocyte transcriptomic profiles from patients with sepsis and healthy controls, was used to evaluate the clinical relevance of Z-DNA binding protein 1 (ZBP1) expression in human sepsis. The mouse infection dataset GSE199546, which contains lung transcriptomic profiles from mice infected with hypervirulent *KP* strain NTUH-K2044 at different time points, was used to assess dynamic host immune responses after *KP* infection.

Raw or processed expression matrices were downloaded from the GEO database and analyzed using R software (version 4.3.3). Differentially expressed genes were identified between the indicated groups according to the thresholds described in the corresponding figure legends. Gene set enrichment analysis (GSEA) was performed to evaluate the enrichment of immune- and inflammation-related pathways. Gene Ontology (GO) and Kyoto Encyclopedia of Genes and Genomes (KEGG) enrichment analyses were performed to identify significantly enriched biological processes and signaling pathways among differentially expressed genes. Adjusted *P*-values were calculated to correct for multiple comparisons, and pathways with adjusted $P<0.05$ were considered significantly enriched.

Animal modeling methods

As previously described, sepsis was induced using the cecal ligation and puncture (CLP) model [1]. Briefly, mice were anesthetized, and a small midline incision was made to expose the cecum. The distal three-quarters of the cecum were ligated with 3–0 silk suture, ensuring no intestinal obstruction occurred. The ligated cecum was then punctured with an 18-gauge needle, and a small amount of fecal material was gently expressed from the puncture site to confirm patency. The incision was then closed. All mice received 0.3 ml of phosphate buffer saline (PBS) post-surgery for fluid resuscitation.

The mouse *KP* infection model was established by intraperitoneal (i.p.) injection of *KP* (NTUH-K2044 or ATCC-700603) under sterile conditions. Briefly, bacterial cultures were grown to the exponential phase, washed three times with sterile PBS, and adjusted to the desired concentration [OD600=1, approximately 5×10^8 colony-forming units (CFU)/ml]. A 29G insulin syringe was used to inject 100 μl of the bacterial suspension into the lower right quadrant of the abdomen, avoiding the midline to prevent organ injury. Control mice received an equal volume of sterile saline. After infection, mice were monitored for clinical symptoms, body weight changes, and survival. At designated time points, blood and tissues were collected for further analysis, including bacterial load quantification, histopathology, and inflammatory cytokine measurements.

At designated time points, mice were euthanized for sample collection, including the intestine, liver, lung, spleen, and peripheral blood. For Western blotting experiments, intestinal, liver, and lung tissues were collected at 12 and 24 h after model induction. For real-time quantitative PCR (qPCR) experiments, intestinal, liver, and lung tissues were collected at 6, 12, and 24 h after model induction. For histopathological analysis, intestinal, liver, and lung tissues were collected 24 h after model induction. For organ bacterial burden assays, blood, liver, lung, and spleen samples were collected 24 h after model induction. Plasma was separated from anticoagulated whole blood by centrifugation at 3000× *g* for 10 min at 4 °C and used for biochemical analysis and enzyme-linked immunosorbent assay (ELISA). Intestinal, liver, and lung tissues were sectioned, snap-frozen in liquid nitrogen, and stored at –80 °C. Tissues from the intestine, liver, and lung were fixed in 4% paraformaldehyde (PFA) for histological analysis. Survival was monitored for up to 14 d. For the RR-11a (HY-112205A; Med Chem Express, USA) injection experiment, RR-11a was administered intraperitoneally at a dose of 20 mg/kg body weight, 6 h before and 12 h after model induction.

RNA isolation and real-time quantitative PCR

RNA was extracted from cells or tissues. Briefly, 1 ml of TRIzol containing the sample was mixed with 200 μl of chloroform. The mixture was centrifuged at 12,000× *g* for 15 min at 4 °C. The upper aqueous phase was carefully collected, discarding the lower phase. The aqueous phase was mixed with 500 μl of isopropanol and incubated for 10 min. RNA was precipitated by centrifugation at 12,000× *g* for 10 min, followed by two washes with 70% ethanol (7500× *g*, 5 min). The RNA pellet was resuspended in RNase-free H_2O.

RNA was then reverse-transcribed into cDNA using the HiScript III RT SuperMix (R323; Vazyme, China) according to the manufacturer's instructions. The resulting cDNA was amplified using SYBR Green QPCR Master Mix (Q711; Vazyme, China) and QuantStudio™ 3 (Applied Biosystems, USA). All samples were analyzed in triplicate.

The data were normalized to β-actin or GAPDH and analyzed using the $2^{-\Delta\Delta Ct}$ method to calculate fold changes. Relative mRNA levels were expressed in arbitrary units, with untreated samples set to 1 for comparison. Primer sequences used in this study are shown in **Additional file 1: Table S1**.

Antibodies

Anti-mouse ZBP1 (mZBP1; AG-20B-0010) antibody was obtained from Adipogen (USA). Anti-human ZBP1 (hZBP1; 13285-1-AP) and anti-signal transducer and activator of transcription 3 (STAT3; 10253-2-AP) antibodies were obtained from Proteintech (USA). Anti-glyceraldehyde-3-phosphate dehydrogenase (GAPDH; A19056), anti-signal transducer and activator of transcription 1 (STAT1; A19563), anti-phospho-STAT1 (p-STAT1; Y701, AP0054), anti-phospho-STAT3 (p-STAT3; Y705, AP0705), anti-BCL2-associated X protein (BAX; A19684), anti-B-cell lymphoma 2 (BCL2; A19693), anti-transcription factor A, mitochondrial (TFAM; A3173), anti-plasminogen activator inhibitor-1 (PAI-1; A6211), anti-Legumain (LGMN; A23776), anti-tissue factor (TF; A23243), and anti-human mixed lineage kinase domain-like protein (hMLKL; A1968) antibodies were obtained from ABclonal (China). Anti-nuclear factor κB P65 (P65; ab32536), anti-phospho-P65 (p-P65; S536, ab76302), anti-Janus kinase 1 (JAK1; ab133666), anti-phospho-JAK1 (p-JAK1; Y1022/Y1023, ab138005), anti-mouse phospho-receptor-interacting serine/threonine-protein kinase 3 (mP-RIPK3; T231/S232, ab22232), anti-mouse phospho-MLKL (mP-MLKL; S345, ab196436), anti-human phospho-RIPK3 (hP-RIPK3; S227, ab209384), and anti-human phospho-MLKL (hP-MLKL; S358, ab18709) antibodies were obtained from Abcam (UK). Anti-mouse fibrin (mFibrin; MABS2155) antibody was obtained from Sigma-Aldrich (USA). Anti-mouse MLKL (mMLKL; 37705), anti-human RIPK3 (hRIPK3; 86671), and anti-mouse RIPK3 (mRIPK3; 15828) antibodies were obtained from Cell Signaling Technology (USA). Anti-Z-DNA (NB100-749) antibody was obtained from Novus Biologicals (USA).

Antibodies used for flow cytometry: Anti-CD86 (3052320), anti-CD206 (2763949), anti-F4/80 (2702204), and anti-CD11b (2513551) antibodies were obtained from Invitrogen (USA).

Cell culture and treatments

Immortalized bone marrow-derived macrophages (iBMDMs), RAW264.7 mouse macrophage-like cells, HEK-293T human embryonic kidney cells, HT29 human colorectal adenocarcinoma cells, A549 human lung adenocarcinoma epithelial cells, and THP-1 human monocytic leukemia cells were obtained from the National Collection of Authenticated Cell Cultures, Chinese Academy of Sciences, China. Peripheral blood mononuclear cells (PBMCs), iBMDMs, HT29, A549, RAW264.7, and HEK-293T cells were cultured *in vitro* in DMEM (G4512; Servicebio, China) supplemented with 10% fetal bovine serum (FBS, 10099141; Gibco, USA) and 100 U/ml penicillin/streptomycin (15140122; Gibco, USA). THP-1 cells were maintained in RPMI 1640 medium (G4532; Servicebio, China) supplemented

with 10% FBS, 100 U/ml penicillin/streptomycin, and 50 μmol β-mercaptoethanol. THP-1 monocytes were differentiated into macrophage-like cells using phorbol 12-myristate 13-acetate (PMA, HY-18739; Med Chem Express, USA). A common method involves incubating THP-1 cells with 50 nmol PMA for 24 h, followed by an additional 24-hour culture in RPMI medium to allow stabilization and maturation into M0 macrophages.

Bone marrow cells were isolated from the femurs and tibias of wild-type (WT), *Zbp1* knockout (*Zbp1*$^{-/-}$), *Ripk3* knockout (*Ripk3*$^{-/-}$), *Mlkl* knockout (*Mlkl*$^{-/-}$), stimulator of interferon genes knockout (*Sting*$^{-/-}$), and interferon alpha/beta receptor 1 knockout (*Ifnar1*$^{-/-}$) mice. The bone marrow was flushed with DMEM and loaded into a 1 ml syringe. Bone marrow cells were cultured at a concentration of 3×10^6/ml in DMEM supplemented with 10% FBS and 20% L929 conditioned medium [or 30 ng/ml macrophage colony-stimulating factor (M-CSF), HY-P70263A; Med Chem Express, USA] to differentiate them into bone marrow-derived macrophages (BMDMs). On day 7, all adherent cells differentiated into BMDMs.

To investigate the role of LGMN, recombinant human LGMN (HY-P70897A; Med Chem Express, USA) and recombinant mouse Lgmn (HY-P70216A; Med Chem Express, USA) were added at a concentration of 50 ng/ml for 1 h before infection, while RR-11a was co-administered with LGMN at a concentration of 50 nmol. In the *KP* (NTUH-K2044 strain and ATCC-700603 strain) infection group, cells were infected at a multiplicity of infection (MOI) of 50:1 for 1 h, followed by three washes with sterile PBS to remove excess bacteria. Gentamicin (100 μg/ml) was then added to eliminate any remaining extracellular bacteria. RNA and protein lysates were collected at specific time points. Mouse recombinant IL-6 (SRP3330; Sigma-Aldrich, USA), STAT3 inhibitor (Stattic, Stat3-In) (HY-13818; Med Chem Express, USA), mouse recombinant IL-4 (HY-P70653; Med Chem Express, USA), mouse recombinant IFN-γ (IF005; Sigma-Aldrich, USA), and lipopolysaccharide (LPS) (L2630; Sigma-Aldrich, USA) were added at final concentrations of 30 ng/ml, 2.5 μmol, 30 ng/ml, 20 ng/ml, and 100 ng/ml respectively. In some experiments, cells were pretreated for 1 h with Stat3-In, 2,2,6,6-tetramethylpiperidin-1-oxyl (TEMPO; HY-112879; Med Chem Express, USA), or N-acetyl cysteine (NAC; HY-B0215; Med Chem Express, USA) at final concentrations of 2.5, 10, and 100 μmol, respectively.

For the co-culture system, BMDMs, iBMDM, and Bend.3, THP-1, HEK-293T, A549, and HT29 cells were seeded in the co-culture chamber (Corning Inc., Corning, USA) following the pattern shown in the schematic diagram in the main figures.

All cells were grown in a 37 °C, 5% CO_2 incubator. The drugs used in the cell experiments were dissolved in Dimethyl sulfoxide (DMSO, HY-Y0320; Med Chem Express, USA).

Immunohistochemical and immunofluorescence staining

The procedures for immunohistochemical (IHC) and immunofluorescence (IF) staining were performed as previously described [1]. Briefly, the intestine, liver, and lung tissue samples were fixed in 4% PFA and embedded in paraffin. Tissue sections on slides were incubated overnight at 4 °C with the indicated primary antibodies [Fibrin (MABS2155, clone 59D8; Sigma-Aldrich, USA); F4/80 (27044-1-AP), CD86 (83523-4-RR), translocase of outer mitochondrial membrane 20 (TOM20) (11802-1-AP), Occludin (27260-1-AP), Claudin-1 (28674-1-AP), LGMN (67017-1-Ig), STAT3 (10253-2-AP), RIPK3 (17563-1-AP), cGAS (26416-1-AP) (Proteintech, USA); ZBP1 (AG-20B-0010-C100; Adipogen, USA)], followed by incubation with the appropriate secondary antibodies. For IHC staining, the antigens were visualized using the peroxidase substrate 3,3'-diaminobenzidine (DAB), resulting in brown labeling of immunoreactive cells. For IF staining, nuclei were counterstained with 4',6-diamidino-2-phenylindole (DAPI; G1012; Servicebio, China). Slides were dried and mounted using ProLong Antifade mounting medium (G1401; Servicebio, China) and visualized using a Nikon fluorescence microscope or a Zeiss LSM 980 laser scanning confocal microscope.

Histological examination

To evaluate intestinal injury, Chiu's score system was used as previously described [1]. To assess pulmonary inflammation and injury, the entire lung surface was systematically evaluated based on the following histopathological parameters: 1) alveolar capillary congestion, 2) inflammatory cell infiltration (neutrophils), 3) presence of hyaline membranes, 4) proteinaceous debris filling the airspaces, and 5) alveolar septal thickening. Each parameter was scored on a scale from 0 (absent) to 2 (severe), and the composite lung injury score was calculated accordingly. The extent of lung injury was evaluated on a semi-quantitative scale of 0–10, defined as follows: 0, not present (normal); 1–4, 10%–40% involvement (mild); 5–6, 50%–60% involvement (moderate); 7–8, 70%–80% involvement (severe); and 9–10, 90%–100% involvement (very severe). For liver injury, histological assessment was performed using hematoxylin and eosin (H&E) stained sections, and the injury score was determined based on: 1) the number of thrombi, 2) the number of (micro)abscesses, 3) the presence and extent of inflammatory infiltration, and 4) the presence and extent of spotty necrosis. All H&E-stained sections of the intestine, lung, and liver were independently evaluated by two experienced pathologists who were blinded to genotype and treatment group allocation.

Enzyme-linked immunosorbent assay

Plasma thrombin-antithrombin complex (TAT), alanine aminotransferase (ALT), aspartate aminotransferase (AST), and creatine kinase (CK) levels were measured in the indicated samples using commercial assay kits: TAT (E-EL-M1138; Elabscience, China), ALT (E-BC-K235-M; Elabscience,

China), AST (E-EL-M0160; Elabscience, China), and CK (E-BC-K558-M; Elabscience, China) according to the manufacturers' instructions.

Transmission electron microscopy

The cultured BMDMs were collected without washing by immediately adding electron microscopy fixative. Cells were gently scraped using a cell scraper and transferred into a centrifuge tube (avoiding trypsin digestion). The cells were centrifuged until a visible pellet, approximately the size of a sesame seed to a mung bean, was obtained. The supernatant was discarded, and fresh electron microscopy fixative was added for fixation at room temperature for 2 h. The sample was then transferred to 4 °C for storage. During storage and transportation, samples were kept at 4 °C with ice packs, ensuring that the fixative did not freeze. Ultrathin sections (70–90 nm) were stained with uranyl acetate and lead citrate, and then examined using an H-7650C transmission electron microscope (Hitachi, Japan).

JC-1 staining for mitochondrial membrane potential in bone marrow-derived macrophages

BMDMs were treated as indicated in each experimental group. After treatment, cells were washed with PBS and incubated with JC-1 staining solution (5 μg/ml in serum-free DMEM; G1515-100T; Servicebio, China) at 37 °C for 20 min. JC-1 forms red fluorescent aggregates in mitochondria with high mitochondrial membrane potential and remains as green fluorescent monomers in depolarized mitochondria with low mitochondrial membrane potential. Cells were then washed twice with JC-1 staining buffer (G1515-100T; Servicebio, China) and imaged using a Zeiss LSM 980 confocal microscope. For quantitative analysis, the ratio of red to green fluorescence intensity was calculated using ImageJ from at least five randomly selected fields per condition; a reduced red/green ratio indicates loss of mitochondrial membrane potential.

RNA interference

The small interfering RNA (siRNAs) used in this study were synthesized by General Biosystems (China). siRNA transfection was performed with 25 nmol siRNA duplexes and 3 μl of Lipofectamine RNAiMAX reagent (13778150; Thermo Fisher Scientific, USA) or INTERFERin® (polyplus-transfection) according to the manufacturer's instructions. The corresponding siRNAs used were as follows: *Scramble*: sense 5'-UUCUCCGAACGUGUCACGUTT-3'; antisense 5'-ACGUGACACGUUCGGAGAATT-3'. *Tfam*: sense 5'-GAGGGAACUUCCUGAUUCATT-3'; antisense 5'-UGAAUCAGGAAGUUCCCUCTT-3′. *Stat3*: sense 5'-GACUUUGAUUUCAACUAUA-3'; antisense 5'-UAUAGUUGAAAUCAAAGUCAAAAAAAAAA-3'.

Flow cytometry

Mature BMDMs infected with *KP* were digested using EDTA, collected, and centrifuged at 800× *g* for 3 min. The supernatant was discarded, and the cell pellet was washed twice with PBS containing 2% FBS. Finally, the cells were resuspended in flow cytometry buffer containing 2% FBS. Specific primary antibodies or staining reagents (including F4/80, CD86, CD206, and CD11b) were added to the cell suspension and incubated for 30 min. The cell pellets were then gently washed with washing buffer 2–3 times to remove unbound antibodies. The washed cell pellets were transferred to sample tubes for flow cytometry. Appropriate parameters and control settings were configured on the flow cytometer. Data analysis and visualization were performed using FlowJo software. To measure mitochondrial contents, cells were stained with MitoSOXTM (M36008; Thermo Fisher Scientific, USA) according to the manufacturer's instructions. Flow cytometry was performed as described above.

RNA sequencing and analysis

Total RNA was extracted from the intestinal tissues of WT and *Zbp1*$^{-/-}$ mice using TRIzol® reagent. The RNA purity was evaluated by measuring the A260/A280 absorbance ratio using a Nanodrop ND-2000 spectrophotometer (Thermo Scientific, USA). The RNA integrity number was determined using an Agilent Bioanalyzer 4150 (Agilent Technologies, USA). Paired-end (PE) libraries were prepared using the ABclonal mRNA-seq Lib Prep Kit following the manufacturer's instructions. mRNA was purified from 1 μg of total RNA using oligo(dT) magnetic beads and fragmented in ABclonal First Strand Synthesis Reaction Buffer. The mRNA fragments were then used as templates to synthesize the first strand of cDNA with random primers and reverse transcriptase (RNase *H*). The second strand of cDNA was synthesized using DNA polymerase *I*, RNase *H*, buffer, and deoxynucleoside triphosphates (dNTPs). The double-stranded cDNA fragments were ligated to adapter sequences for PCR amplification. The PCR products were purified, and the quality of the libraries was evaluated using an Agilent Bioanalyzer 4150. Finally, sequencing was performed on the Illumina NovaSeq 6000 platform.

Evans Blue staining to assess the permeability of the intestine

On day 2 (24 h) after *KP* infection, WT, *Zbp1*$^{-/-}$, *Ripk3*$^{-/-}$, *Mlkl*$^{-/-}$, *Sting*$^{-/-}$, *Zbp1*$^{fl/fl}$*Lyz2-Cre*$^{+/-}$ or myeloid-specific *Lgmn* knockout *(Lgmn*$^{fl/fl}$*Lyz2-Cre*$^{+/-}$*, Lgmn*^{CKO}*)* mice on a C57BL/6 background were randomly selected to evaluate intestinal permeability. Animals were fasted for 6 h prior to the experiment (with free access to water) to minimize the influence of food on EB absorption. Sixty minutes before sacrifice, mice were injected via the tail vein with 2% (w/v) EB solution in PBS at a dose of 1 ml/kg.

After euthanasia, a 0–10 cm ileal segment proximal to the cecum was excised and gently rinsed with pre-cooled PBS to remove surface blood and mucus. One portion of the intestine was laid flat (mucosal side up) on white filter paper or a Petri dish, kept moist, and photographed. The remaining tissue was

weighed, homogenized, and lysed in N,N-dimethylformamide (DMF) at 4 μl/mg of tissue. The lysates were incubated at 55 °C for 48 h, followed by centrifugation at 12,000× *g* for 10 min at 4 °C. The absorbance of the supernatant was measured at 620 nm.

Standard EB solutions (0.1, 1, 5, 10, 25, and 50 μg/ml in PBS) were prepared to generate a calibration curve, which was used to calculate the amount of EB extravasation in intestinal tissues, thereby reflecting intestinal barrier permeability.

Transepithelial electrical resistance assessment

Transepithelial electrical resistance (TEER) was measured using a Millicell ERS-2 volt-ohm meter (Merck Millipore, USA). HT29 and A549 cells were seeded in the upper chamber of the Transwell system. For A549 and HT29 cells, successful establishment of the Transwell cell model was confirmed when nearly 100% confluence was observed under a microscope and TEER values stabilized between 600 and 800 $\Omega \cdot cm^2$. To monitor TEER changes, measurements were taken every 15 min after stabilization until the TEER value reached its lowest point. TEER was then assessed at 0, 6, 12, and 24 h after treatments to evaluate barrier integrity dynamics.

Transcription factor-target gene prediction

Potential upstream transcription factors of LGMN were predicted using the hTFtarget database (http://swisstargetprediction.ch/), a database of human transcription factor-target gene regulatory relationships. *LGMN* was used as the input target gene, and candidate transcription factors predicted to regulate LGMN were retrieved. Among the 149 candidate ZBP1-interacting proteins, only STAT1 and STAT3 were predicted to be upstream transcriptional regulators of LGMN*,* and STAT3 was selected for further experimental validation.

Prediction of STAT3-binding motifs in the *LGMN* promoter

Potential STAT3-binding motifs in the promoter regions of mouse *Lgmn* and human *LGMN* were predicted using the JASPAR database (https://jaspar.elixir.no/). Briefly, the promoter sequences upstream of the transcription start sites of mouse *Lgmn* and human *LGMN* were retrieved from the corresponding reference genome sequences. The STAT3 position frequency matrix was obtained from JASPAR and used to scan the promoter regions for putative STAT3-binding sites. Candidate binding motifs with high prediction scores were selected and visualized to show the potential STAT3-binding sequences in the mouse *Lgmn* and human *LGMN* promoters.

Chromatin immunoprecipitation assay

Chromatin immunoprecipitation (ChIP) was performed using a standard magnetic bead-based protocol (Servicebio, China) to assess the binding of STAT3 to the *Lgmn* promoter in *KP*-infected macrophages. Briefly, cells were cross-linked with 1% formaldehyde at 37 °C for 10 min, and crosslinking was

quenched with 125 mmol glycine for 5 min at room temperature. After washing with ice-cold PBS, the cells were lysed in IP lysis buffer containing protease inhibitors, and chromatin was sonicated using a JY92-IIN ultrasonic processor (Ningbo Xinyi, China) at 30% amplitude (4.5 s on/10.5 s off, 20 cycles) on ice to yield DNA fragments of approximately 200–500 bp.

The lysates were centrifuged at 12,000× *g* for 10 min at 4 °C, and the supernatants were precleared with Protein A/G agarose beads. The chromatin was immunoprecipitated overnight at 4 °C with 1 μg of anti-STAT3 antibody (10253-2-AP; Proteintech, USA) or normal rabbit IgG. Protein A/G beads were added and incubated for an additional 2 h at 4 °C with rotation. After sequential washes with low-salt, high-salt, LiCl, and TE buffers, the immune complexes were eluted and subjected to reverse cross-linking with 0.2 mmol NaCl and 20 μg proteinase K at 55 °C overnight. DNA was purified using a magnetic bead-based DNA purification kit.

Purified DNA was analyzed by qPCR using SYBR Green Master Mix and primers specific to the predicted STAT3-binding site within the *Lgmn* promoter region (sense: 5’-AAGGCCAGCCTAGTCCACAG-3’; antisense: 5’-ATTCAGATTTCAACCCCACA-3’). PCR conditions were as follows: 95 °C for 10 min, followed by 40 cycles of 95 °C for 15 s and 60 °C for 60 s. Data were normalized to input chromatin and expressed as fold enrichment over the IgG.

Dual luciferase reporter assay system

To assess STAT3-dependent transcriptional regulation of *Lgmn*, we performed a dual-luciferase reporter assay. The mouse *Lgmn* promoter region upstream of the transcription start site was cloned into pGL1-SV40-RLuc-ΔPro-hLuc+(firefly) to generate reporter constructs (Corues Biotechnology, China). A full-length fragment (−3000 bp to the CDS) and serial truncations corresponding to P1 (–2000 bp to CDS), P2 (–1200 bp to CDS), and P3 (–800 bp to CDS) were constructed (Corues Biotechnology, China); all plasmids were prepared endotoxin-free and sequence-verified. pcDNA3.1-STAT3 (or pcDNA3.1 empty vector) was used for transcription-factor overexpression, and pGL1-SV40-RLuc served as the Renilla internal control. Experimental groupings followed the design file (empty reporter±STAT3; *Lgmn*-promoter reporter±STAT3).

HEK-293T cells were seeded into 24-well plates (70%–80% confluence at transfection) and co-transfected with the indicated firefly reporter, pcDNA3.1-STAT3 (or vector control), and the Renilla control using a liposomal transfection reagent according to the manufacturer’s protocol. At 24–48 h post-transfection, cells were lysed, and firefly and Renilla activities were measured sequentially with a dual-luciferase kit on a luminometer. Firefly signals were normalized to Renilla to yield relative luciferase activity (Luc/Rluc). Data were analyzed from at least three independent experiments.

Mass spectroscopy

BMDMs from different groups were washed with PBS and subjected to immunoprecipitation using an anti-ZBP1 antibody. The immunoprecipitates were separated by SDS-PAGE and stained with Coomassie Brilliant Blue. The gels were then washed twice and stained at room temperature for 30 min. After destaining and reduction, the gel bands were digested with trypsin at 37 °C for 16 h. The resulting peptide extracts were combined with the supernatants from each gel spot and completely dried.

Samples were reconstituted in nano-HPLC buffer, loaded onto a trap column, and separated on an RP-C18 analytical column at 300 nl/min. Peptides were analyzed on an LTQ Orbitrap Velos Pro mass spectrometer in positive ion mode using a 105-min run. MS data were acquired over an m/z range of 350-1800 after instrument calibration. For data-dependent acquisition, the top 15 most intense precursor ions from each full scan were selected for CID fragmentation. MS scans were acquired at a resolution of 60,000, and proteins were identified with a confidence interval of at least 95% using the MASCOT v2.3 search engine (Matrix Science Ltd., UK).

Reference

1. Wu J, Liu Q, Zhang X, Tan M, Li X, Liu P, *et al.* The interaction between STING and NCOA4 exacerbates lethal sepsis by orchestrating ferroptosis and inflammatory responses in macrophages. Cell Death Dis. 2022;13(7):653. https://doi.org/10.1038/s41419-022-05115-x.

Table S1 RT-qPCR primers used in this study

Gene	Forward primer sequence (5'–3')	Reverse primer sequence (5'–3')
hZBP1	AACATGCAGCTACAATTCCAGA	AGTCTCGGTTCACATCTTTTGC
mZbp1	AAGAGTCCCCTGCGATTATTTG	TCTGGATGGCGTTTGAATTGG
hGAPDH	ACAACTTTGGTATCGTGGAAGG	GCCATCACGCCACAGTTTC
mActin	GGCTGTATTCCCCTCCATCG	CCAGTTGGTAACAATGCCATGT
mGapdh	CATCACTGCCACCCAGAAGACTG	ATGCCAGTGAGCTTCCCGTTCAG
mIfit1	TACAGGCTGGAGTGTGCTGAGA	CTCCACTTTCAGAGCCTTCGCA
mIfit3	GCTCAGGCTTACGTTGACAAGG	CTTTAGGCGTGTCCATCCTTCC
mIrf4	GAACGAGGAGAAGAGCGTCTTC	GTAGGAGGATCTGGCTTGTCGA
mIrf5	CCTACAGAACCACTCTTGCCTG	CCTTGTGGGTTGCTGATGGTGA
mIrf7	CCTCTGCTTTCTAGTGATGCCG	CGTAAACACGGTCTTGCTCCTG
mIl6	TACCACTTCACAAGTCGGAGGC	CTGCAAGTGCATCATCGTTGTTC
hLGMN	GGACGTGGAAGATCTGACTAAA	CTGGCTTTGCGTTTCATACC
mLgmn	AGAGGATGTGACTCCAGAGAA	CCGTGGTCGGTGAAGTAAAT

RT-qPCR. Reverse transcription quantitative real-time polymerase chain reaction; hZBP1. Human Z-DNA binding protein 1; mZbp1. Mouse Z-DNA binding protein 1; hGAPDH. Human glyceraldehyde-3-phosphate dehydrogenase; mActin. Mouse actin; mGapdh. Mouse glyceraldehyde-3-phosphate dehydrogenase; mIfit1. Mouse interferon-induced protein with tetratricopeptide repeats 1; mIfit3. Mouse interferon-induced protein with tetratricopeptide repeats 3; mIrf4. Mouse interferon regulatory factor 4; mIrf5. Mouse interferon regulatory factor 5; mIrf7. Mouse interferon regulatory factor 7; mIl6. Mouse interleukin-6; hLGMN. Human Legumain; mLgmn. Mouse Legumain

Figure legends

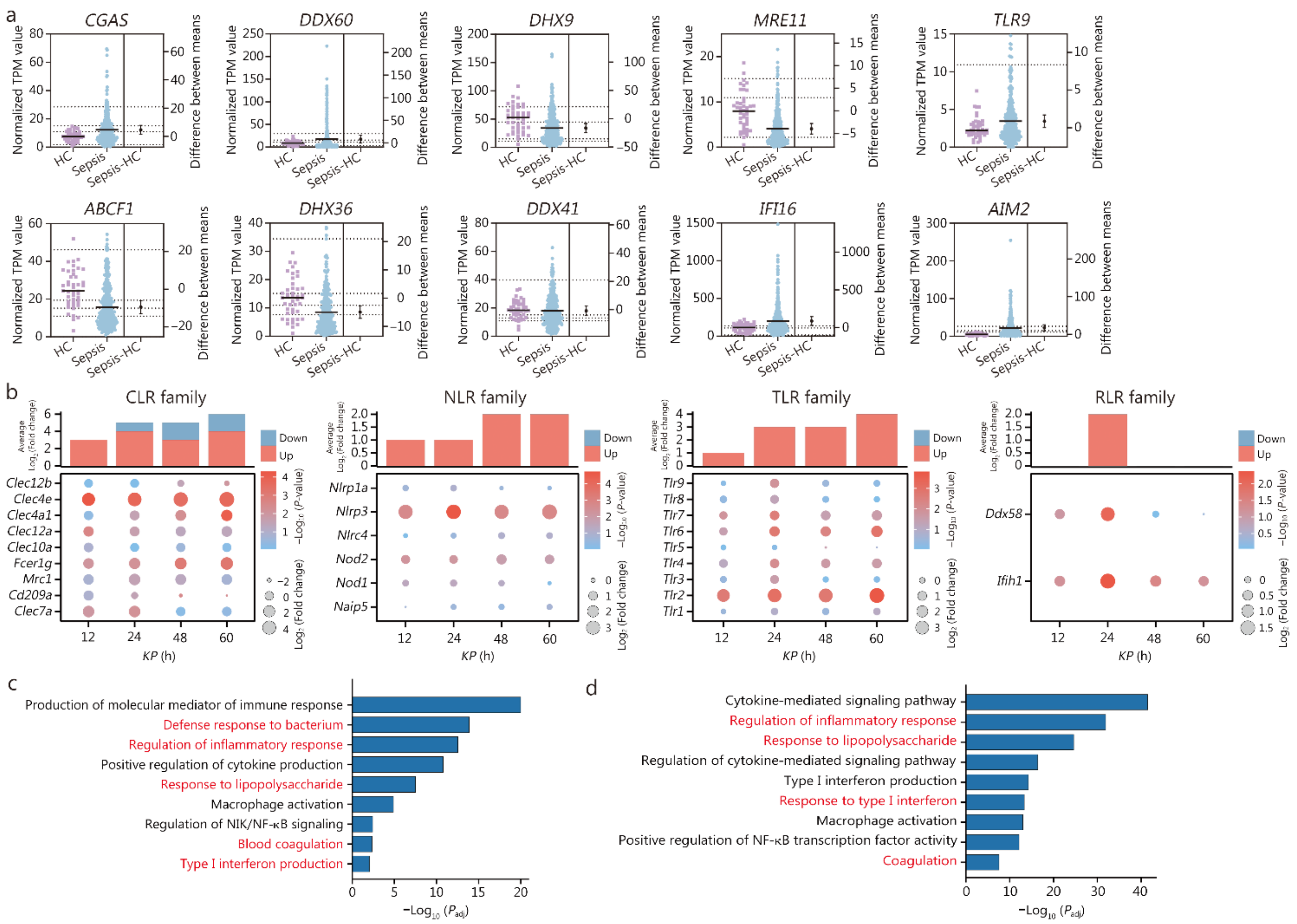

Fig. S1 Expression patterns of nucleic acid sensors and innate immune pathways in public datasets of human sepsis and *KP* infection. **a** Comparison of DNA sensor expression levels in peripheral blood between sepsis patients in dataset GSE185263 (n=348) and healthy controls (HC, n=44). Statistical significance was assessed separately using the Mann-Whitney U test. **b** Heatmap of differential gene expression of *KP*-infected mice in dataset GSE199546. Dynamic changes in pattern recognition receptors (PRRs) from the CLR, NLR, TLR, and RLR families were compared in lung tissues at 12, 24, 48, and 60 h after *KP* infection with non-infection. **c** Pathway enrichment analysis based on lung transcriptomic profiling of peripheral blood (HC vs. sepsis). **d** Pathway enrichment analysis based on lung transcriptomes from mice infected with *KP* (0 h vs. 24 h). HC. Healthy control; CGAS. Cyclic GMP-AMP synthase; DDX60. DExD/H-box helicase 60; DHX9. DExH-box helicase 9; MRE11. MRE11 homolog, double-strand break repair nuclease; TLR9. Toll-like receptor 9; ABCF1. ATP-binding cassette subfamily F member 1; DHX36. DEAH-box helicase 36; DDX41. DExD-box helicase 41; IFI16. Interferon gamma inducible protein 16; AIM2. Absent in melanoma 2; Clec12b. C-type lectin domain family 12 member B; Clec4e. C-type lectin domain family 4 member E; Clec4a1. C-type lectin domain family 4 member A1; Clec12a. C-type lectin domain family 12 member A; Clec10a. C-type lectin domain family 10 member A; Fcer1g. Fc epsilon receptor Ig; Mrc1. Mannose receptor C-type 1; Cd209a. CD209 antigen-like protein A; Clec7a. C-type lectin domain family 7 member A;

Nlrp1a. NLR family pyrin domain containing 1A; Nlrp3. NLR family pyrin domain containing 3; Nlrc4. NLR family CARD domain containing 4; Nod2. Nucleotide-binding oligomerization domain containing 2; Nod1. Nucleotide-binding oligomerization domain containing 1; Naip5. NLR family apoptosis inhibitory protein 5; Tlr9. Toll-like receptor 9; Tlr8. Toll-like receptor 8; Tlr7. Toll-like receptor 7; Tlr6. Toll-like receptor 6; Tlr5. Toll-like receptor 5; Tlr4. Toll-like receptor 4; Tlr3. Toll-like receptor 3; Tlr2. Toll-like receptor 2; Tlr1. Toll-like receptor 1; Ddx58. DExD/H-box helicase 58; Ifih1. Interferon induced with helicase C domain 1

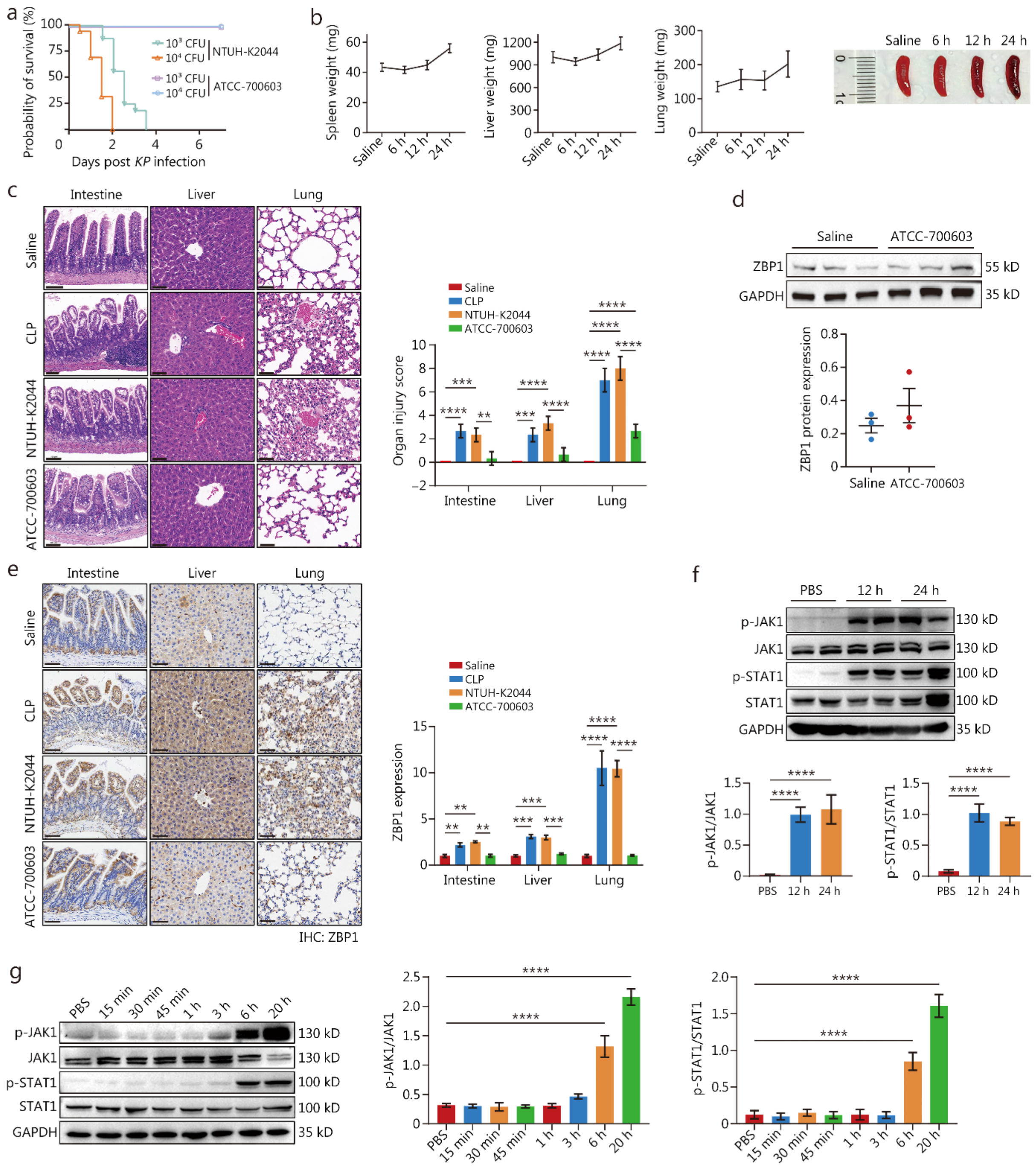

Fig. S2 NTUH-K2044 induces more severe sepsis-associated injury and ZBP1 activation than ATCC-700603. **a** Kaplan-Meier survival analysis of mice infected with NTUH-K2044 and ATCC-700603 strains (*n*=16). Survival differences were analyzed using the log-rank (Mantel-Cox) test. **b** Assessment of spleen, liver, and lung weight in mice infected with the NTUH-K2044 strain (4000 CFU) at 6, 12, and 24 h, and spleen injury image (*n*=6). Data are shown as mean±SD. **c** H&E staining and histopathological scoring of intestinal, liver, and lung tissues from mice infected with the ATCC-700603 and NTUH-K2044 strains (*n*=3). For the intestine, scale bar=100 μm. For the liver and lung, scale bar=50 μm. **d** Detection of ZBP1 protein levels in the lungs of mice infected with the ATCC-700603 strain (1000 CFU). **e** Immunohistochemical detection of ZBP1 in the intestine, liver, and lungs of infected mice (*n*=3). For the intestine, scale bar=100 μm. For the liver and lung, scale bar=50 μm. **f**

Analysis of JAK1-STAT1 signaling pathway activation in BMDMs following infection with the NTUH-K2044 strain (MOI=50:1). **g** Analysis of JAK1-STAT1 signaling pathway activation in THP-1 cells following infection with the NTUH-K2044 strain (MOI=50:1). Data are shown as mean±SD. Statistical significance was assessed using the Kruskal-Wallis test followed by Dunn's multiple comparisons test (**c, e**), the Unpaired two-tailed Student's *t*-test (**d**), and the One-way ANOVA followed by Dunnett's multiple comparisons test (**f, g**). $^{**}P<0.01$, $^{***}P<0.001$, $^{****}P<0.0001$. CFU. Colony-forming unit; *KP*. *Klebsiella pneumoniae*; CLP. Cecal ligation and puncture; ZBP1. Z-DNA binding protein 1; GAPDH. Glyceraldehyde-3-phosphate dehydrogenase; IHC. Immunohistochemistry; JAK1. Janus kinase 1; STAT1. Signal transducer and activator of transcription 1; PBS. Phosphate-buffered saline

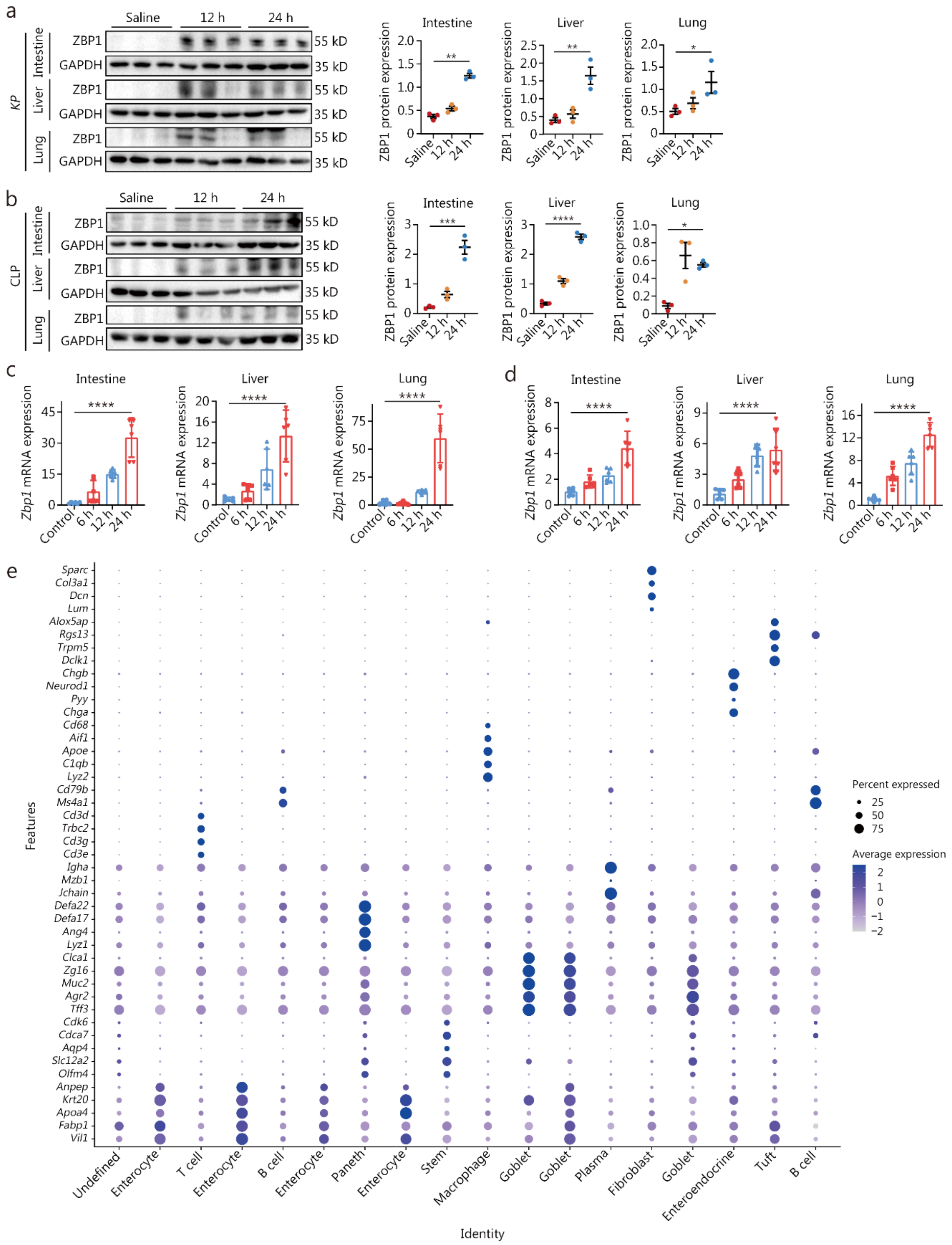

Fig. S3 ZBP1 expression in multi-organ sepsis models and intestinal single-cell populations. ZBP1 protein levels in intestinal, liver, and lung tissues of *KP*- **(a)** and CLP **(b)**-induced sepsis mice (*n*=3). Quantitative analysis of protein expression levels. *Zbp1* mRNA expression in the intestine, liver, and lung tissues of *KP*- (**c**) or CLP (**d**)-induced sepsis mice (*n*=6). **e** Dot plot of marker genes defining cell clusters from single-cell RNA sequencing of intestinal tissues in septic mice. Data are shown as mean±SD. Statistical significance was assessed using one-way ANOVA followed by Dunnett's multiple comparisons test (**a-d**). $^{*}P<0.05$, $^{**}P<0.01$, $^{***}P<0.001$, $^{****}P<0.0001$. *KP*. *Klebsiella*

pneumoniae; CLP. Cecal ligation and puncture; ZBP1. Z-DNA binding protein 1; GAPDH. Glyceraldehyde-3-phosphate dehydrogenase; Sparc. Secreted protein acidic and rich in cysteine; Col3a1. Collagen type III alpha 1 chain; Dcn. Decorin; Lum. Lumican; Alox5ap. Arachidonate 5-lipoxygenase activating protein; Rgs13. Regulator of G protein signaling 13; Trpm5. Transient receptor potential cation channel subfamily M member 5; Dclk1. Doublecortin-like kinase 1; Chgb. Chromogranin B; Neurod1. Neuronal differentiation 1; Pyy. Peptide YY; Chga. Chromogranin A; Aif1. Allograft inflammatory factor 1; Apoe. Apolipoprotein E; C1qb. Complement C1q B chain; Lyz2. Lysozyme 2; Cd79b. Cluster of differentiation 79B; Ms4a1. Membrane-spanning 4-domains subfamily A member 1; Cd3d. Cluster of differentiation 3 delta; Trbc2. T cell receptor beta constant 2; Cd3g. Cluster of differentiation 3 gamma; Cd3e. Cluster of differentiation 3 epsilon; Igha. Immunoglobulin heavy constant alpha; Mzb1. Marginal zone B and B1 cell-specific protein; J chain. Joining chain of multimeric IgA and IgM; Defa22. Defensin alpha 22; Defa17. Defensin alpha 17; Ang4. Angiogenin 4; Lyz1. Lysozyme 1; Clca1. Chloride channel accessory 1; Zg16. Zymogen granule protein 16; Muc2. Mucin 2; Agr2. Anterior gradient 2; Tff3. Trefoil factor 3; Cdk6. Cyclin-dependent kinase 6; Cdca7. Cell division cycle associated 7; Aqp4. Aquaporin 4; Slc12a2. Solute carrier family 12 member 2; Olfm4. Olfactomedin 4; Anpep. Alanyl aminopeptidase; Krt20. Keratin 20; Apoa4. Apolipoprotein A4; Fabp1. Fatty acid binding protein 1; Vil1. Villin 1

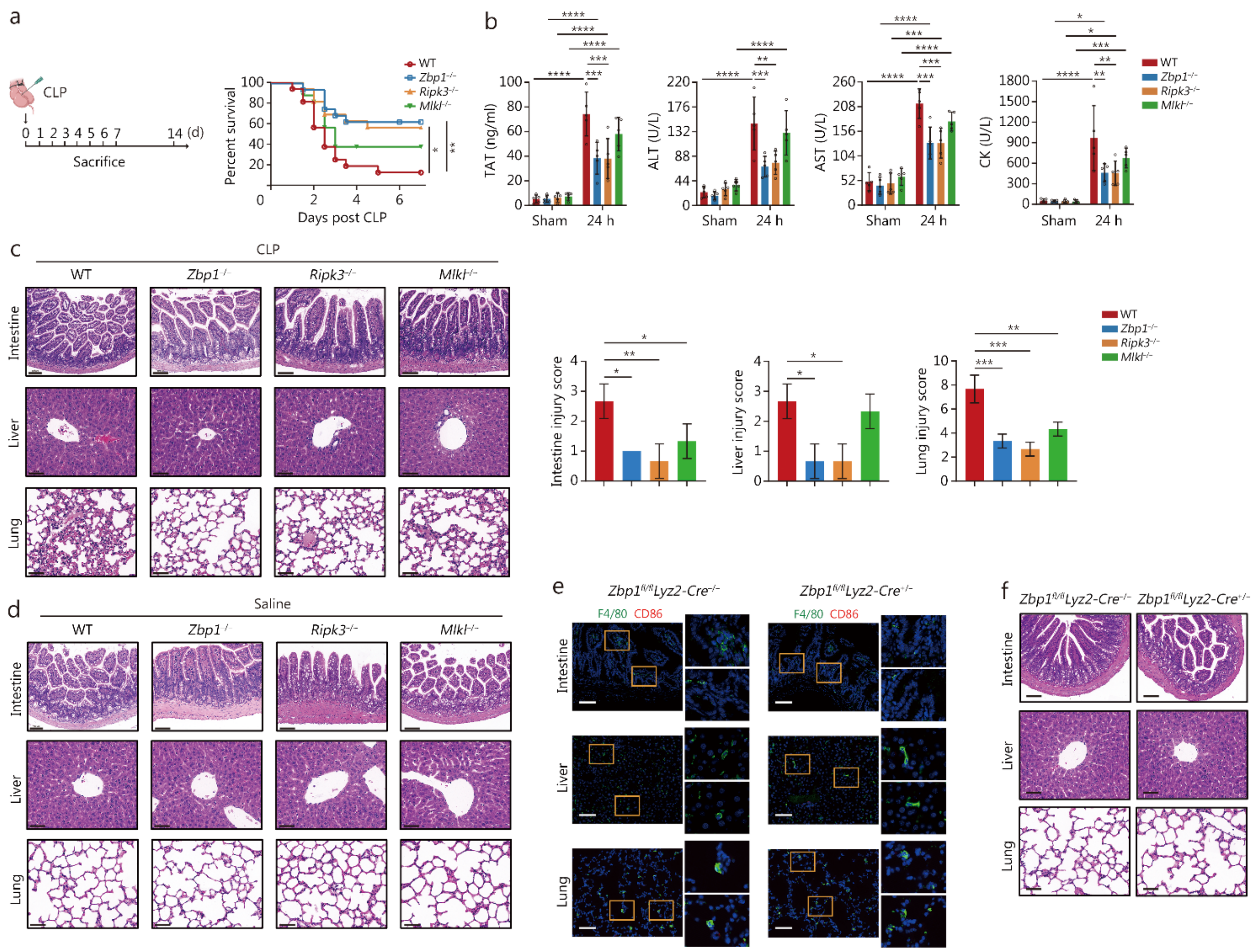

Fig. S4 *Zbp1* deficiency ameliorates CLP-induced sepsis and organ injury. **a** Schematic overview of the experimental modeling timeline. Kaplan-Meier survival curves of CLP-operated mice with different genotypes (*n*=16). Survival differences were analyzed using the log-rank (Mantel-Cox) test. **b** Serum levels of TAT, ALT, AST, and CK at 24 h post-sepsis CLP (*n*=5). **c** H&E staining and histopathological assessment of intestinal, liver, and lung tissues from CLP-operated (24 h) (*n*=3). For the intestine, scale bar=100 μm. For the liver and lung, scale bar=50 μm. **d** Representative H&E staining sections of intestine, liver, and lung tissues from WT, *Zbp1*$^{-/-}$, *Ripk3*$^{-/-,}$ and *Mlkl*$^{-/-}$ mice under basal (saline) conditions (*n*=3). For the intestine, scale bar=100 μm. For the liver and lung, scale bar=50μm. **e** Immunofluorescence (IF) analysis of macrophage activation in intestinal, liver, and lung tissues from *Zbp1*$^{fl/fl}$*Lyz2-Cre*$^{-/-}$ and *Zbp1*$^{fl/fl}$*Lyz2-Cre*$^{+/-}$ mice under basal (saline) conditions (*n*=3). Representative images show staining for the macrophage marker F4/80 (green), the activation marker CD86 (red), and nuclei (DAPI, blue). For the intestine, scale bar=100 μm. For the liver and lung, scale bar=50 μm. **f** H&E staining sections of the intestine, liver, and lung from *Zbp1*$^{fl/fl}$*Lyz2-Cre*$^{-/-}$ and *Zbp1*$^{fl/fl}$*Lyz2-Cre*$^{+/-}$ mice maintained under basal (saline) conditions (*n*=3). For the intestine, scale bar=100 μm. For the liver and lung, scale bar=50 μm. Data are shown as mean±SD. Statistical significance was assessed using two-way ANOVA followed by Tukey's multiple comparisons test (**b**) and the Kruskal-Wallis test followed by Dunn's multiple comparisons test (**c**). $^{*}P$<0.05, $^{**}P$<0.01,

$^{***}P<0.001$, $^{****}P<0.0001$. CLP. Cecal ligation and puncture; ZBP1. Z-DNA binding protein 1; RIPK3. Receptor-interacting protein kinase 3; MLKL. Mixed lineage kinase domain-like protein; TAT. Thrombin-antithrombin complex; ALT. Alanine transaminase; AST. Aspartate aminotransferase; CK. Creatine kinase

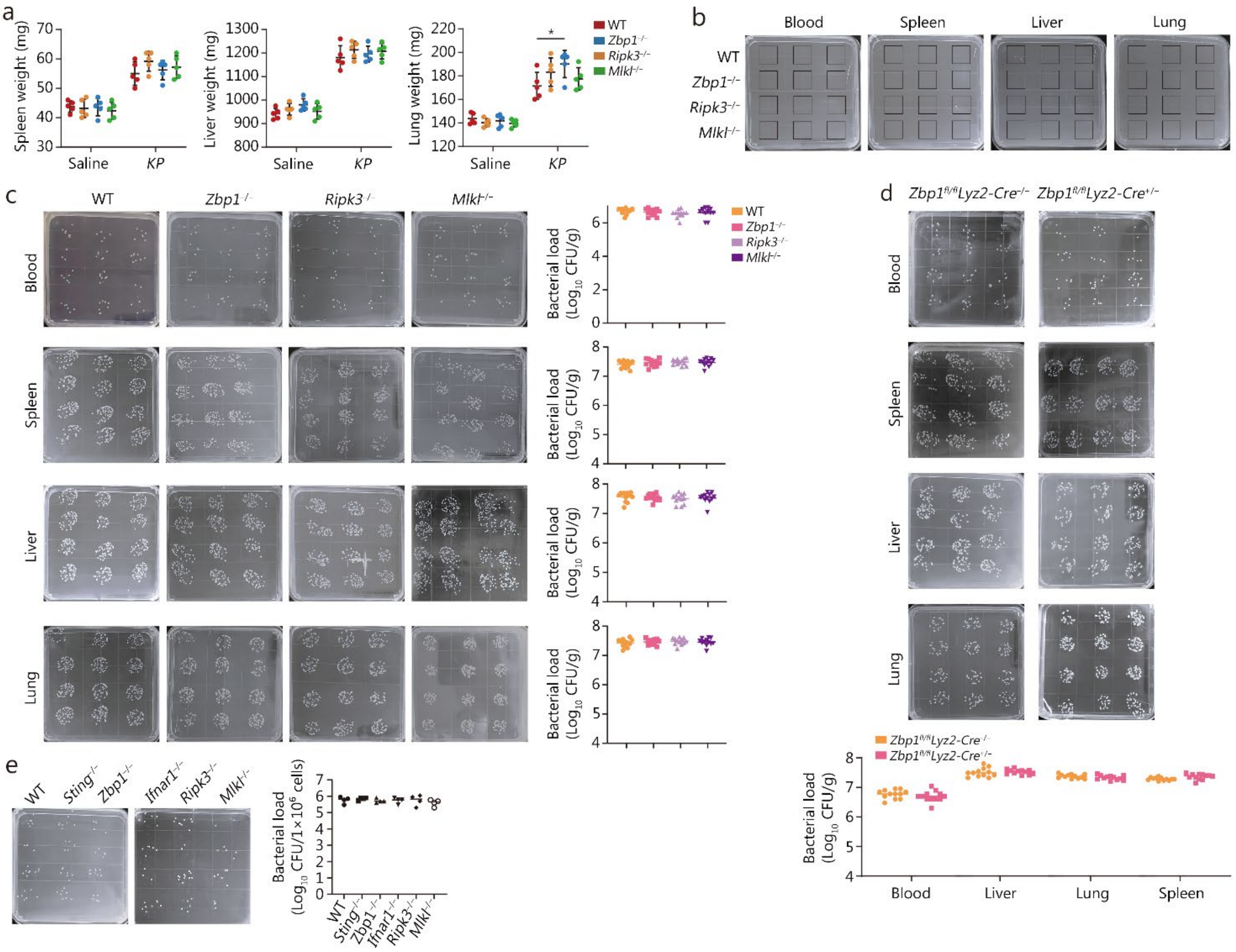

Fig. S5 Comparable bacterial burdens in different genotypes of mice and macrophages following *KP* infection. **a** Spleen, liver, and lung weights of WT, *Zbp1*$^{-/-}$, *Ripk3*$^{-/-}$, and *Mlkl*$^{-/-}$ mice at 24 h after *KP* infection or saline injection (*n*=5). No significant differences were observed among genotypes. **b** Bacterial colonies plated from homogenized blood, spleen, liver, and lung of WT, *Zbp1*$^{-/-}$, *Ripk3*$^{-/-}$, and *Mlkl*$^{-/-}$ mice after saline injection, showing no bacterial growth in controls (*n*=3). **c** Colony-forming unit (CFU) assays from blood, spleen, liver, and lung homogenates of *KP*-infected WT, *Zbp1*$^{-/-}$, *Ripk3*$^{-/-}$, and *Mlkl*$^{-/-}$ mice at 24 h post-infection (*n*=12). Quantification revealed no significant differences in bacterial loads between genotypes. **d** Bacterial CFU counts in blood, spleen, liver, and lung homogenates from *Zbp1*$^{fl/fl}$*Lyz2-Cre*$^{-/-}$ and *Zbp1*$^{fl/fl}$*Lyz2-Cre*$^{+/-}$ mice after *KP* infection (*n*=12). **e** Intracellular bacterial counts in WT, *Sting*$^{-/-}$, *Zbp1*$^{-/-}$, *Ifnar1*$^{-/-}$, *Ripk3*$^{-/-}$, and *Mlkl*$^{-/-}$ BMDMs 6 h after *KP* infection (*n*=4). Data are shown as mean±SD. Statistical significance was assessed using the Two-way ANOVA followed by Tukey's multiple comparisons test (**a**), the One-way ANOVA followed by Dunnett's multiple comparisons test (**c, e**), and the Unpaired two-tailed Student's *t*-test (**d**). $^{*}P<0.05$,. *KP*. *Klebsiella pneumoniae*; ZBP1. Z-DNA binding protein 1; RIPK3. Receptor-interacting protein kinase 3; MLKL. Mixed lineage kinase domain-like protein; STING. Stimulator of interferon genes; IFNAR1. Interferon alpha/alpha receptor 1

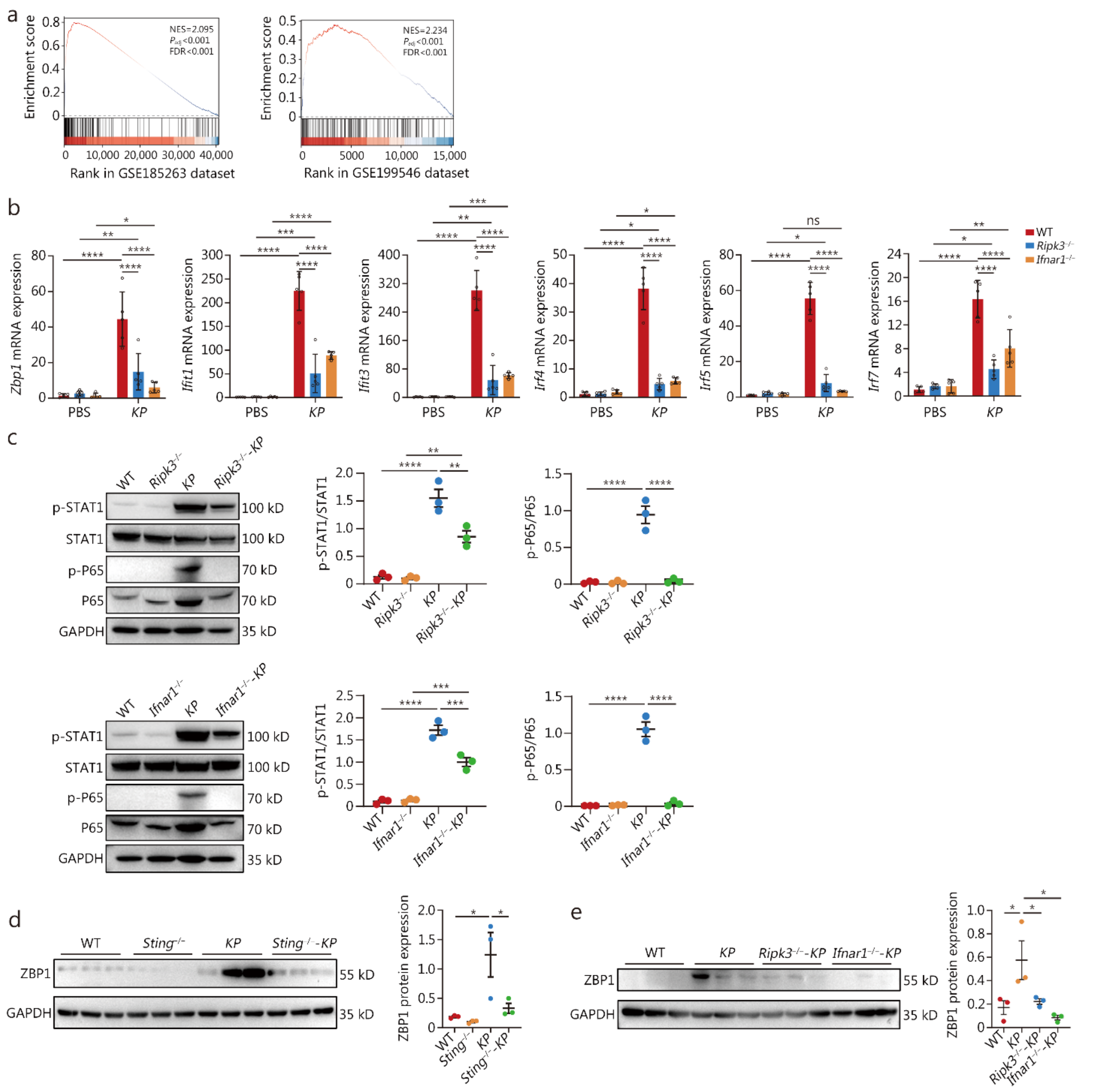

Fig. S6 ZBP1 activation in macrophages during *KP* infection depends on type I interferon (IFN-I) signaling. **a** GSEA analysis of interferon signaling pathways based on differentially expressed genes from the GSE185263 and GSE199546 datasets. **b** Expression of interferon-stimulated genes, including *Zbp1*, *Ifit1*, *Ifit3*, *Irf4*, *Irf5* and *Irf7*, in BMDMs of different genotypes after *KP* infection in WT, *Ripk3*$^{-/-}$ and *Ifnar1*$^{-/-}$ BMDMs (MOI=50:1, 6 h) (*n*=5). **c** Western blotting confirming the suppression of classical downstream inflammatory signaling pathways of INF-I after *KP* infection in WT, *Ripk3*$^{-/-}$ and *Ifnar1*$^{-/-}$ BMDMs (MOI=50:1, 6 h). **d** Western blotting confirming the dependence of ZBP1 induction on *Sting*$^{-/-}$ in the intestine from mice with *KP* infection (4000 CFU, 24 h) (*n*=3). **e** Western blotting confirming the dependence of ZBP1 induction on *Ripk3*$^{-/-}$ and *Ifnar1*$^{-/-}$ in the intestine from mice with *KP* infection (4000 CFU, 24 h) (*n*=3). Data are shown as mean±SD. Statistical significance was assessed using the two-way ANOVA followed by Tukey's multiple comparisons test (**b**), the One-way ANOVA followed by Dunnett's multiple comparisons test (**c**), and the Kruskal-Wallis test followed

by Dunn's multiple comparisons test (**d, e**). $^{*}P$<0.05, $^{**}P$<0.01, $^{***}P$<0.001, $^{****}P$<0.0001. *KP*. *Klebsiella pneumoniae*; Zbp1. Z-DNA binding protein 1; Ifit1. Interferon-induced protein with tetratricopeptide repeats 1; Ifit3. Interferon-induced protein with tetratricopeptide repeats 3; Irf4. Interferon regulatory factor 4; Irf5. Interferon regulatory factor 5; Irf7. Interferon regulatory factor 7; RIPK3. Receptor-interacting protein kinase 3; STING. Stimulator of interferon genes; IFNAR1. Interferon alpha/beta receptor 1. STAT1. Signal transducer and activator of transcription 1; P65. Nuclear factor κB p65; GAPDH. Glyceraldehyde-3-phosphate dehydrogenase

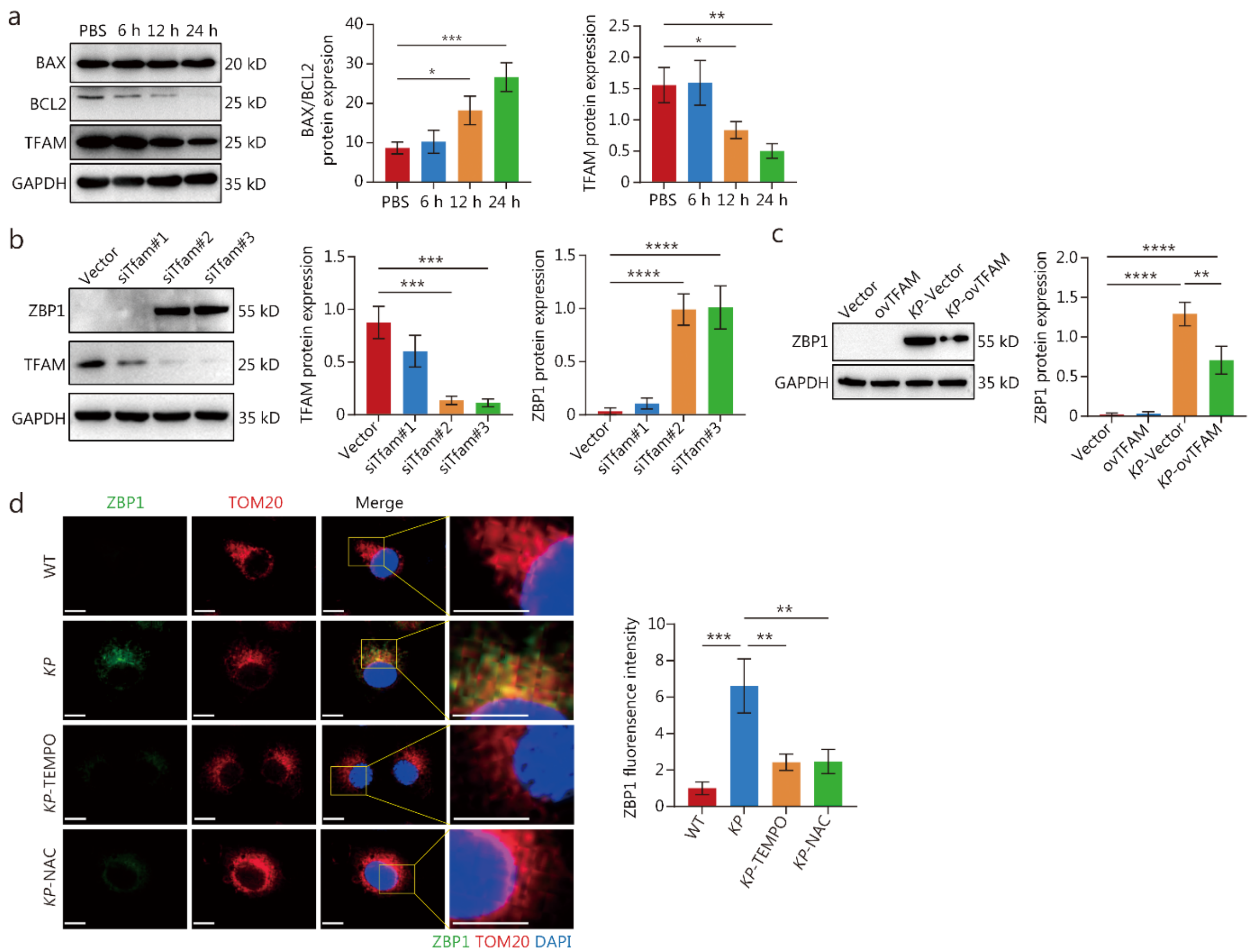

Fig. S7 Mitochondrial damage in macrophages induced by *KP* infection activates ZBP1. **a** Expression of mitochondrial damage-associated proteins in BMDMs following *KP* infection (MOI=50:1) (*n*=3). **b** Western blotting analysis of ZBP1 and TFAM expression in immortalized bone marrow-derived macrophages (iBMDMs) transfected with vector or *Tfam*-targeting siRNAs (siTfam#1–#3) (*n*=3). **c** Western blotting analysis of ZBP1 expression in iBMDMs transfected with a TFAM-overexpression plasmid (ovTFAM) or vector (*n*=3). **d** Representative confocal immunofluorescence images showing ZBP1 (green) and the mitochondrial marker TOM20 (red) in BMDMs from WT mice under PBS or following *KP* infection (MOI=50:1, 6 h). WT macrophages pre-treated with mitochondrial ROS scavengers TEMPO (10 μmol, 1 h) or NAC (100 μmol, 1 h), followed by *KP* infection (MOI=50:1, 6 h), are also shown (*n*=3). Scale bars=5 μm. Data are presented as mean±SD. Statistical significance was assessed by the Kruskal-Wallis test followed by Dunn's multiple comparisons test (**a-c**) and the one-way ANOVA followed by Dunnett's multiple comparisons test (**d**). $^{*}P<0.05$, $^{**}P<0.01$, $^{***}P<0.001$, $^{****}P<0.0001$. BAX. BCL2-associated X protein; BCL2. B-cell lymphoma 2; TFAM. Transcription factor A, mitochondrial; GAPDH. Glyceraldehyde-3-phosphate dehydrogenase; ZBP1. Z-DNA binding protein 1; TOM20. Translocase of outer mitochondrial membrane 20; DAPI. 4',6-diamidino-2-phenylindole; PBS. Phosphate-buffered saline; *KP*. *Klebsiella pneumoniae*; TEMPO. 2,2,6,6-tetramethylpiperidine-1-oxyl; NAC. N-acetylcysteine; siTfam. Small interfering RNA targeting Tfam; ovTFAM. TFAM overexpression; WT. Wild type

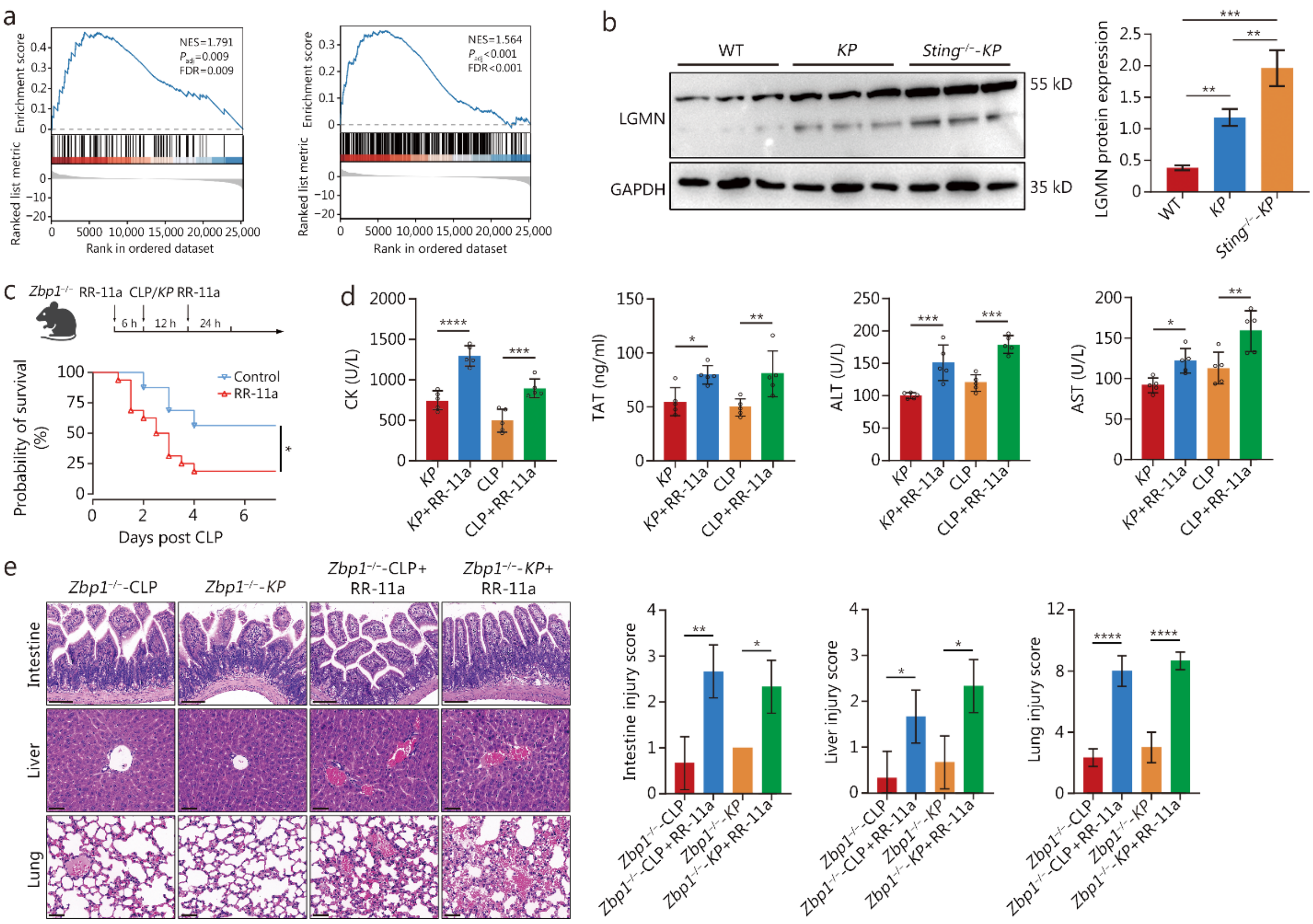

Fig. S8 LGMN mediates the protective effects of *Zbp1* deficiency against *KP*-induced sepsis. **a** GSEA enrichment analysis of lipid metabolism pathways based on differentially expressed intestinal genes between *Zbp1*$^{-/-}$ and WT mice after *KP* infection (4000 CFU; 24 h, *n*=3). **b** Western blotting analysis of LGMN expression in intestinal tissues from WT and *Sting*$^{-/-}$ mice following *KP* infection (4000 CFU, 24 h). LGMN induction was attenuated in *Sting*$^{-/-}$ mice (*n*=3). **c** Schematic of the experimental design involving RR-11a (10 mg/kg) treatment in *KP*-infected (4000 CFU) or CLP-operated *Zbp1*$^{-/-}$ mice. Kaplan-Meier survival analysis of CLP-operated *Zbp1*$^{-/-}$ mice treated with RR-11a via intraperitoneal injection (*n*=16). Survival differences were analyzed using the log-rank (Mantel-Cox) test. **d** Measurement of serum CK, TAT, ALT, and AST levels in *Zbp1*$^{-/-}$ mice treated with RR-11a following *KP* infection or CLP (*n*=5). **e** H&E staining and histological injury scoring of intestine, liver, and lung tissues in *Zbp1*$^{-/-}$ mice subjected to *KP* (4000 CFU; 24 h) infection or CLP (24 h), with or without RR-11a (10 mg/kg) treatment (*n*=3). For the intestine, scale bar=100 µm. For the liver and lung, scale bar=50 µm. Data are shown as mean±SD. Statistical significance was assessed using the Kruskal-Wallis test followed by Dunn's multiple comparisons test (**b, e**) and the unpaired two-tailed Student's *t*-test (**d**). $^{*}P$<0.05, $^{**}P$<0.01, $^{***}P$<0.001, $^{****}P$<0.0001. NES. Normalized enrichment score; P_{adj}. Adjusted *P*-value; FDR. False discovery rate; WT. Wild type; *KP*. *Klebsiella pneumoniae*; STING. Stimulator of interferon genes; LGMN. Legumain; GAPDH. Glyceraldehyde-3-phosphate dehydrogenase; ZBP1. Z-DNA binding protein 1; CLP. Cecal ligation and puncture; CK. Creatine

kinase; TAT. Thrombin-antithrombin complex; ALT. Alanine aminotransferase; AST. Aspartate aminotransferase

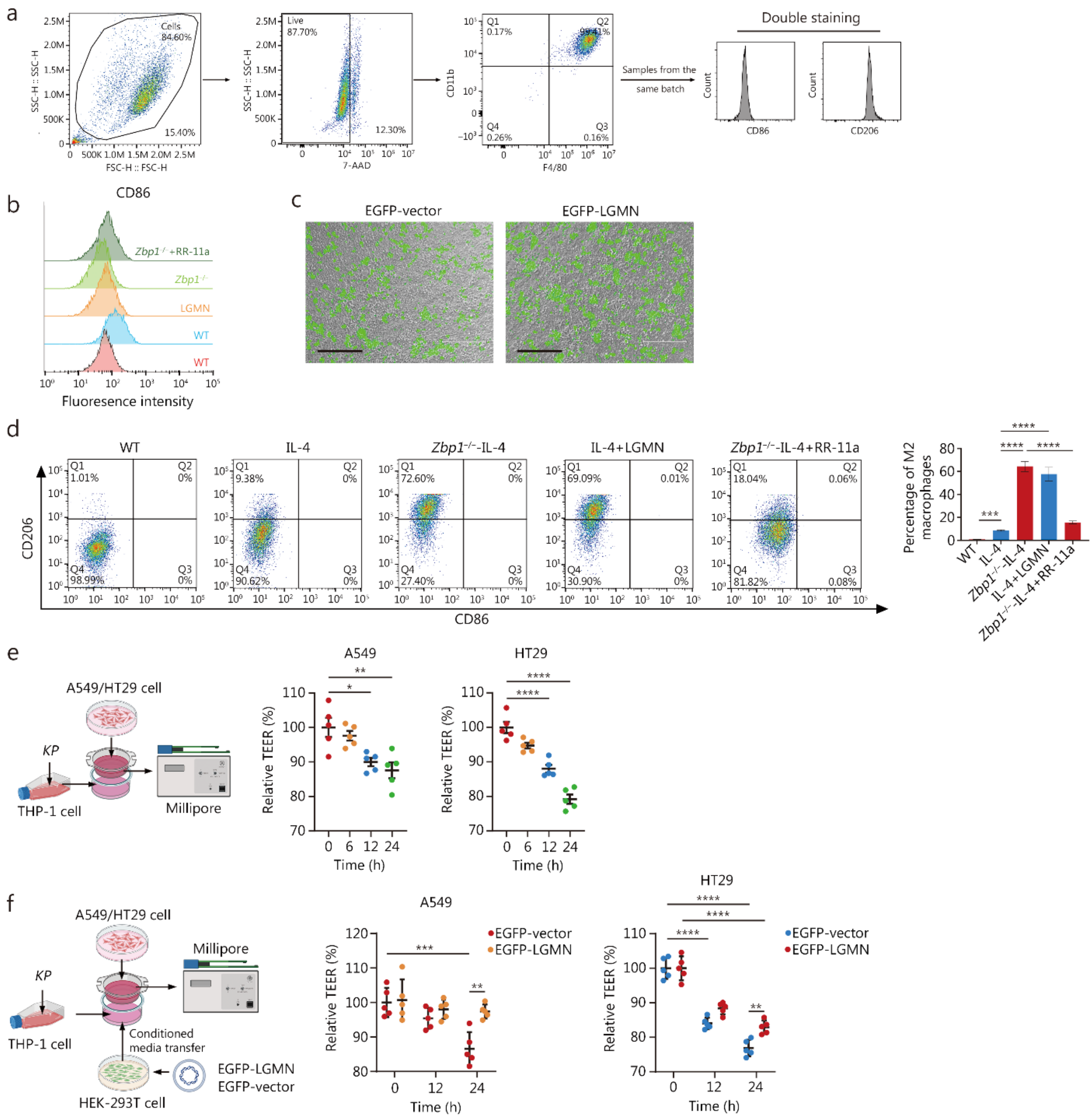

Fig. S9 Deletion of *Zbp1* suppresses the proinflammatory state of macrophages by upregulating LGMN. **a** Representative flow cytometry plots showing the sequential gating strategy used for macrophage analysis. **b** Experimental showing treatment of WT and *Zbp1*$^{-/-}$ BMDMs under M1 polarization conditions [LPS (100 ng/ml) + mIFN-γ (20 ng/ml), 12 h] with LGMN (50 ng/ml) or its inhibitor RR-11a (50 nmol/L). Flow cytometry analysis of CD86 expression in WT and *Zbp1*$^{-/-}$ BMDMs under different conditions (*n*=5). **c** Conditioned medium transfer from LGMN-overexpressing HEK-293T cells to THP-1 cells, followed by *KP* infection (MOI=50:1, 12 h). Fluorescence microscopy showing differential expression levels of the two plasmids. **d** Flow cytometry analysis of CD206 and CD86 expression in mIL-4-treated (30 ng/ml, 24 h) BMDMs with or without LGMN (50 ng/ml) or RR-11a (50 nmol/L) (*n*=3). **e** Schematic diagram of the co-culture experimental design. THP-1 cells were infected with *KP* (MOI=50:1) for 12 h prior to co-culture. TEER measurements were recorded starting at 0 h of co-culture. A549 AND HT29 cells had formed

confluent monolayers prior to co-culture. Disruption of epithelial barrier integrity in A549 and HT29 monolayers following co-culture with infected THP-1 cells. **f** Schematic diagram of co-culture of A549/HT29 cells with THP-1 and supernatants from HEK-293T cells transfected with EGFP-LGMN. THP-1 cells were infected with *KP* (MOI=50:1) for 12 h prior to co-culture, and HEK-293T supernatants were added 1 h before co-culture. TEER measurements were recorded from 0 h of co-culture. A549 and HT29 cells were pre-grown to confluence. Supernatant-derived LGMN from HEK-293T cells mitigated epithelial barrier damage induced by co-culture with THP-1 cells (n=5). Data are shown as mean±SD. Statistical significance was assessed using the Kruskal-Wallis test followed by Dunn's multiple comparisons test (**d**), the One-way ANOVA followed by Dunnett's multiple comparisons test (**e**), and the Two-way ANOVA followed by Tukey's multiple comparisons test (**f**). $^{*}P<0.05$, $^{**}P < 0.01$, $^{***}P<0.001$, $^{****}P<0.0001$. FSC-H. Forward scatter height; SSC-H. Side scatter height; LPS. Lipopolysaccharide; IFN-γ. Interferon-γ; ZBP1. Z-DNA binding protein 1; LGMN. Legumain; WT. Wild type; EGFP. Enhanced green fluorescent protein; *KP*. *Klebsiella pneumoniae*; IL-4. Interleukin-4; TEER. Transepithelial electrical resistance

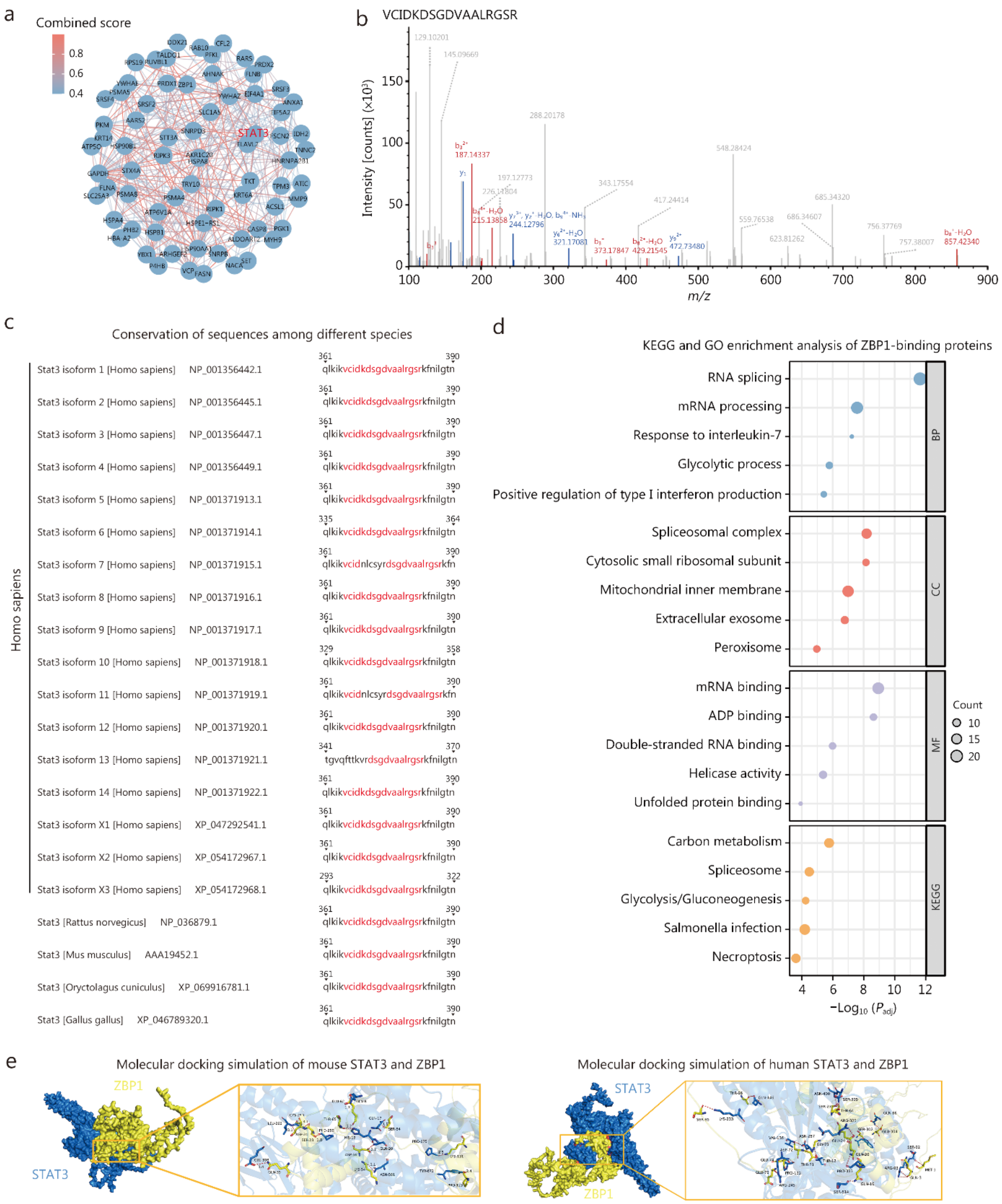

Fig. S10 ZBP1 interacts with STAT3. **a** Protein-protein interaction (PPI) network analysis of ZBP1-interacting proteins. **b, c** Evolutionarily conserved binding (V365-R382) sites between STAT3 and ZBP1 in the *KP*-infected BMDM. **d** KEGG and GO enrichment analysis of proteins interacting with ZBP1. **e** Protein-protein docking simulation of human and mouse ZBP1 binding to STAT3. STAT3. Signal transducer and activator of transcription 1; ZBP1. Z-DNA binding protein 1

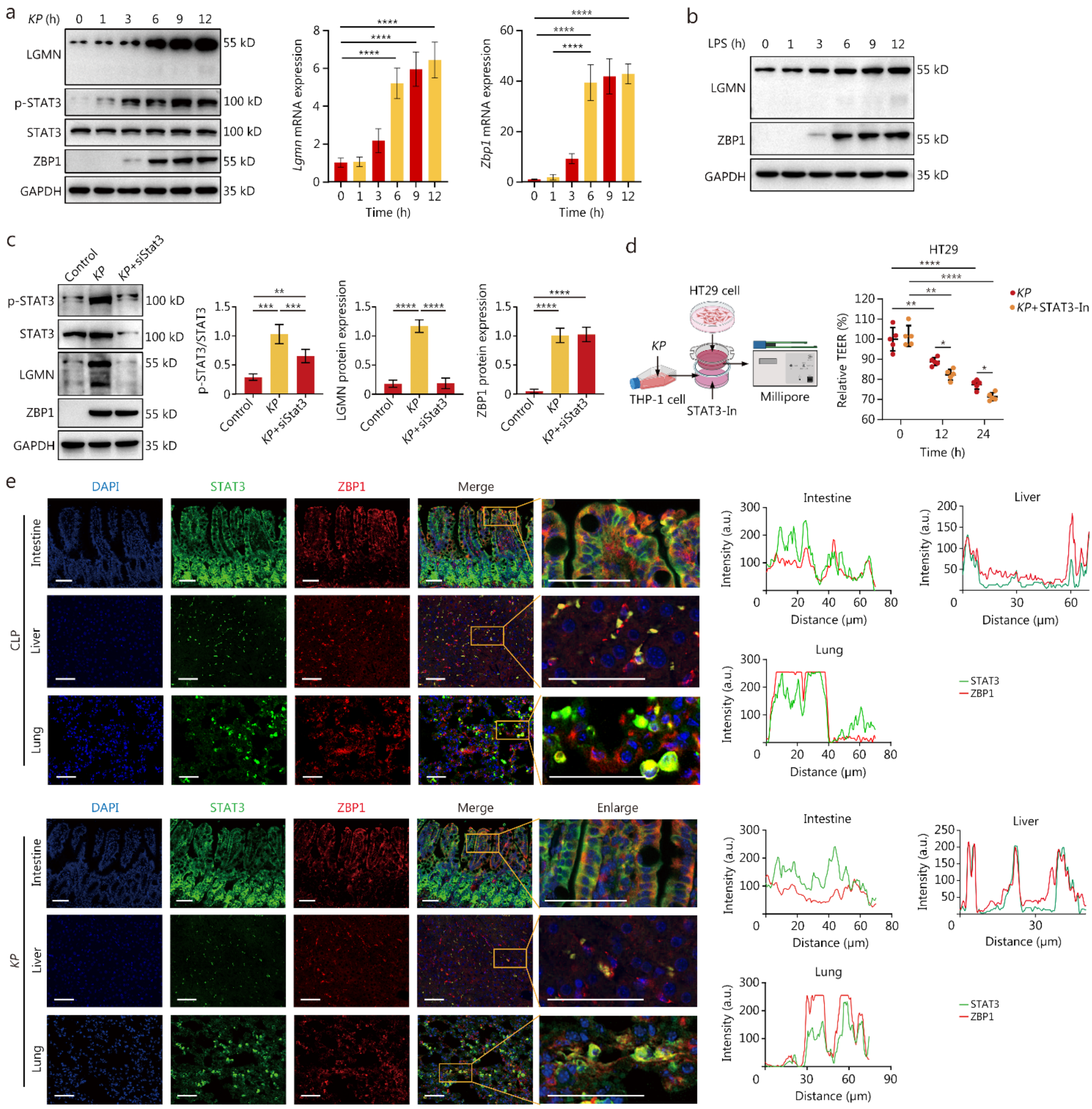

Fig. S11 *KP*-induced ZBP1 suppresses STAT3-dependent LGMN expression and contributes to epithelial barrier dysfunction. **a** BMDMs were infected with *KP* at a multiplicity of infection (MOI) of 50:1 for the indicated times (0, 1, 3, 6, 9, and 12 h) (*n*=5). Protein levels of LGMN, p-STAT3, STAT3, and ZBP1 were detected by Western blotting, with GAPDH used as the loading control. The mRNA levels of *Zbp1* and *Lgmn* were measured by qRT-PCR. **b** BMDMs were stimulated with LPS (100 ng/ml) for the indicated times (0, 1, 3, 6, 9, and 12 h). **c** Western blotting analysis of P-STAT3, STAT3, LGMN, and ZBP1 (*n*=3). **d** THP-1 cells were infected with *KP* (MOI=50:1) for 12 h prior to co-culture. RR-11a (50 nmol/L), LGMN (50 ng/ml), and STAT3 inhibitor (Stattic, Stat3-In) (2.5 μmol/L) were added 1 h prior to co-culture. TEER measurements were recorded starting at 0 h of co-culture (*n*=5). **e** Representative immunofluorescence images showing DAPI (blue), STAT3 (green), and ZBP1 (red) staining in the intestine, liver, and lung from mice subjected to CLP (12 h) or *KP*

infection (4000 CFU, 12 h) (n=3). Merged images and higher-magnification views (boxed areas) illustrate the spatial distribution and partial colocalization of ZBP1 and STAT3 *in situ.* Data are shown as mean±SD. Statistical significance was assessed using the One-way ANOVA followed by Dunnett's multiple comparisons test (**a**), the Kruskal-Wallis test followed by Dunn's multiple comparisons test (**c**), and the Two-way ANOVA followed by Tukey's multiple comparisons test (**d**). $^{*}P<0.05$, $^{**}P<0.01$, $^{***}P<0.001$, $^{****}P<0.0001$. *KP*. *Klebsiella pneumoniae*; LPS. Lipopolysaccharide; LGMN. Legumain; p-STAT3. Phosphorylated signal transducer and activator of transcription 3; STAT3. Signal transducer and activator of transcription 3; ZBP1. Z-DNA binding protein 1; GAPDH. Glyceraldehyde-3-phosphate dehydrogenase; siStat3. Small interfering RNA targeting Stat3; TEER. Transepithelial electrical resistance; CLP. Cecal ligation and puncture; DAPI. 4',6-diamidino-2-phenylindole

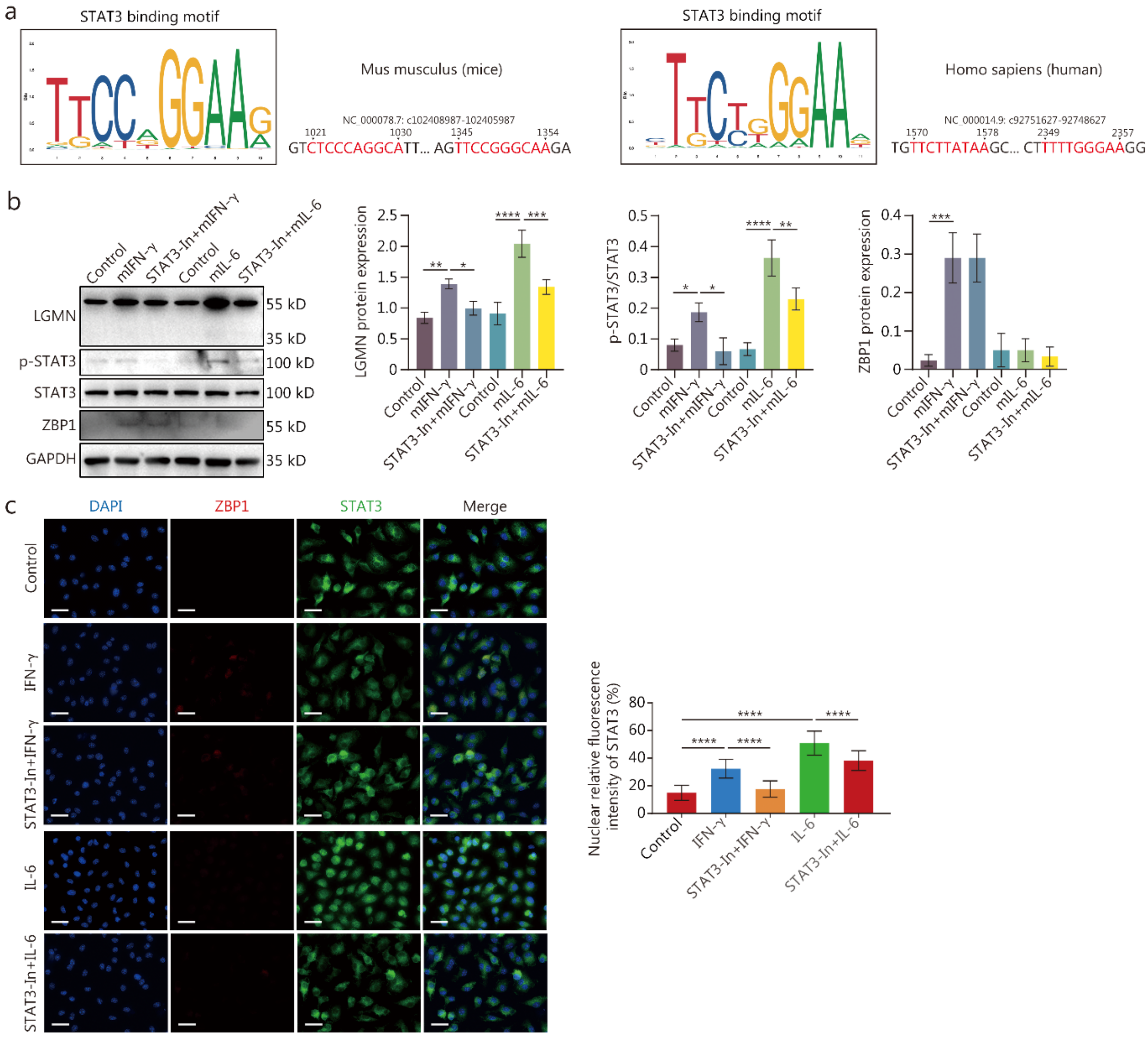

Fig. S12 ZBP1 inhibits the nuclear translocation of STAT3 to suppress LGMN transcription. **a** Alignment of human and mouse STAT3-binding sequences within the *LGMN* promoter region using the JASPAR database. **b** BMDMs were stimulated with recombinant murine IFN-γ (20 ng/ml, 6 h) and IL-6 (30 ng/ml, 6 h), and the protein levels of LGMN, p-STAT3, STAT3, ZBP1, and GAPDH were analyzed (n=3). **c** Immunofluorescence staining of STAT3 and ZBP1 in BMDMs stimulated with IFN-γ (20 ng/ml, 6 h) or IL-6 (30 ng/ml, 6 h). Scale bar=50 μm. Each group randomly selected 15 cells. Data are shown as mean±SD. Statistical significance was assessed using the Kruskal-Wallis test followed by Dunn's multiple comparisons test (**b**), and the One-way ANOVA followed by Dunnett's multiple comparisons test (**c**). $^{*}P<0.05$, $^{**}P<0.01$, $^{***}P<0.001$, $^{****}P<0.0001$. STAT3. Signal transducer and activator of transcription 3; LGMN. Legumain; p-STAT3. Phosphorylated signal transducer and activator of transcription 3; ZBP1. Z-DNA binding protein 1; GAPDH. Glyceraldehyde-3-phosphate dehydrogenase; DAPI. 4',6-diamidino-2-phenylindole; IFN-γ. Interferon-γ; IL-6. Interleukin-6
